# Supplementary material for: Influence of N-Methylation and Conformation on Almiramide Anti-Leishmanial Activity
Source: Molecules. 2021 Jun 12;26(12):3606. doi: 10.3390/molecules26123606 (PMC8231256; doi:10.3390/molecules26123606)
Supplement: Supplementary file 1 [file molecules-26-03606-s001.zip › molecules-1250988-supplementary.pdf]

Supporting informations

Influence of *N*-methylation and conformation on almiramide anti-leishmanial activity

Anh Minh Thao Nguyen[†], Skye Brettel[†], Noélie Douanne,[£] Claudia Duquette,[£] Audrey Corbeil,[£] Emanuella F. Fajardo[‡], Martin Olivier,[‡] Christopher Fernandez-Prada,[£] William D. Lubell[†]*

[†]Départements de chimie et [£]de pathologie et microbiologie, Université de Montréal, C.P. 6128, Succursale Centre-Ville, Montréal, Québec, H3C3J7, Canada and [‡]Department of Microbiology and Immunology, McGill University, Montréal, Canada

Table of contents

Synthesis procedure of Agl and Nai dipeptides……………………………………………………S2

NMR Spectra……………………………………………………………………………………….S7

Ascertainment of purity by HPLC…………………………………………………………...S14

Dose-response curves of *Leishmania* strains and LM-1 macrophage survival rate in the presence of peptides 12-37……………………………………………………………………………..S31

Synthesis procedure of Agl and Nai dipeptides

Fmoc-Agl dipeptides were synthesized as described for the synthesis of Fmoc-Agl-Val-OH in reference 36 in the article. In brief, Fmoc-Agl-Ala-OH and Fmoc-Agl-Phe-OH were prepared by alkylation of the corresponding *N*-Boc-methionyl dipeptide *tert*-butyl ester with CH_3I, lactam cyclization using NaH to provide *N*-(Boc)Agl dipeptide *tert*-butyl esters **39b-c**, removal of the acid labile protection, and amine protection with Fmoc-OSu giving Fmoc-Agl dipeptide acids **38b-c**. Characterization of Boc-Agl dipeptide *tert*-butyl ester **39b-c**, and Fmoc-Agl dipeptide acids **38b-c** are presented below.

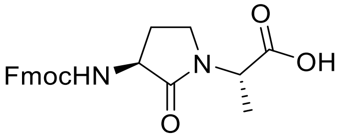

38b

***N*-Fmoc-(3*S*)-Agl-(2*S*)-Ala-OH (38b)** : R*f* 0.23 (10% MeOH in DCM); $[\alpha]_D^{25}$ –43° (*c* 1.5, MeOH); ^{1}H NMR (500 MHz, $CDCl_3$) δ 9.58 (brs, 2H), 7.74 (d, *J* = 7.5 Hz, 2H), 7.58 (m, 2H), 7.38 (t, *J* = 7.5 Hz, 2H), 7.30 – 7.26 (m, 2H), 4.86 – 4.72 (m, 1H), 4.52 – 4.43 (m, 1H), 4.36 (m, 2H), 4.20 (m, 1H), 3.51-3.46 (m, 1H), 3.41 – 3.30 (m, 1H), 2.57 (m, 1H), 2.07 – 1.93 (m, 1H), 1.45 (d, *J* = 7.0 Hz, 3H); ^{13}C NMR (126 MHz, $CDCl_3$) δ 174.1, 173.9, 156.9, 143.7, 141.3, 127.8, 127.2, 125.2, 120.1, 67.6, 52.8, 50.4, 47.1, 41.7, 27.3, 14.5. HRMS (ESI$^+$) calcd *m/z* for $C_{22}H_{23}N_2O_5$ $[M+H]^+$, 395.1601 found 395.1602.

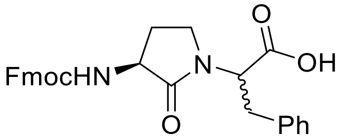

38c

***N*-Fmoc-(3*S*)-Agl-(2*R*,*S*)-Phe-O-*t*-Bu (38c)** was isolated as a 3:2 mixture that was assessed based on the integration of the phenylalanine α-proton signals at 4.99 and 4.95 ppm. Proton and carbon NMR signals of the minor diastereoisomer are written respectively in brackets and parentheses. R*f* 0.41 (30% EtOAc in hexane); ^{1}H NMR (500 MHz, $CDCl_3$) δ 7.30 – 7.24 (m, 4H), 7.24 – 7.20 (m,

1H), [7.18 – 7.16 (m, 4H)], [4.99 (dd, J = 11.3, 6.4 Hz, 1H)], 4.95 (dd, *J* = 11.3, 5.4 Hz, 1H), [4.15 (br s, 1H)], 3.94 (br s, 1H), 3.43 (t, *J* = 9.1 Hz, 1H), [3.38 – 3.24 (m, 2H)], 3.17 (m, 1H), [3.07 (m, 1H)], 3.02 – 2.93 (m, 2H), 2.57 – 2.50 (m, 2H), [1.44 (s, 9H)], 1.43 (s, 9H), 1.42 (s, 9H); ^{13}C NMR (126 MHz, $CDCl_3$) δ 173.6, 173.3, 156.6, 143.9, (143.8), 141.4, (141.3), 136.5, (136.4), 128.9, 128.8, 128.5, 127.8, (127.7), 127.2, 125.3, 120.1, 67.2, (53.0), 55.2, 47.1, (52.2), 42.1, 34.9, 27.6, 27.4, (27.8); HRMS (ESI$^+$) calcd *m/z* for $C_{22}H_{33}N_2O_5$ [M+H]$^+$ 405.2384, found 405.2392.

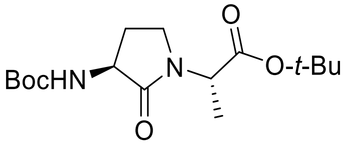

39b

***N*-Boc-(3*S*)-Agl-(2*S*)-Ala-O-*t*-Bu (39b)** : R_f 0.35 (30% EtOAc in hexane); $[\alpha]_D^{25}$ –68° (*c* 1.2, MeOH); ^{1}H NMR (500 MHz, $CDCl_3$) δ 5.10 (s, 1H), 4.72 (q, *J* = 7.4 Hz, 1H), 4.24 (br s, 1H), 3.46 (t, *J* = 8.9 Hz, 1H), 3.31 (td, *J* = 9.7, 6.4 Hz, 1H), 2.71-2.62 (m, 1H), 1.89 (d, *J* = 10.7 Hz, 1H), 1.45 (s, 18H), 1.38 (d, *J* = 7.5 Hz, 3H); δ ^{13}C NMR (126 MHz, $CDCl_3$) δ 172.5, 170.4, 156.1, 82.1, 80.0, 52.5, 50.5, 41.1, 29.0, 28.5, 28.1, 15.1. HRMS (ESI$^+$) calcd *m/z* for $C_{16}H_{29}N_2O_5$ [M+H]$^+$, 329.2071 found 329.2074.

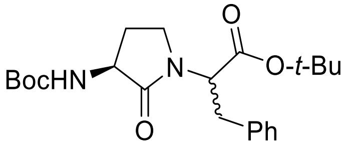

39c

***N*-Boc-(3*S*)-Agl-(2*R*,*S*)-Phe-OH (39c)** was isolated as a 2:1 mixture that was assessed based on the integration of the phenylalanine α-proton signals at 5.07 and 4.88 ppm. Signals of the minor diastereoisomer are written respectively in brackets and parentheses. R_f 0.30 (10% MeOH in DCM); ^{1}H NMR (500 MHz, $CDCl_3$) δ 7.85 – 7.66 (m, 2H), 7.59 (m, 2H), 7.40 (m, 2H), 7.30 (m, 4H), 7.26 – 7.22 (m, 1H), 7.19 (m, 2H), 5.93 (br s, 1H), [5.57 (br s, 1H)], 5.07 (dd, *J* = 11.1, 4.0 Hz, 1H), [4.88 (m, 1H)], 4.40 – 4.33 (m, 1H), [4.31 - 4.26 (m, 1H)], 4.22 – 4.18 (m, 1H), [4.13 – 4.07 (m, 1H)], 3.50 – 3.39 (m, 2H), 3.29 – 3.13 (m, 2H), 3.03 (dd, *J* = 14.8, 11.8 Hz, 1H), 2.46 (br s, 1H), 1.95 – 1.83 (m, 1H), [1.68 – 1.59 (m, 1H)]; ^{13}C NMR (126 MHz, $CDCl_3$) δ (172.9), 172.7,

169.3, (169.1), 155.9, (155.4), 136.5, (136.1), 128.7, (128.6), 128.5, (128.4), 127.0, (126.9), 82.4, (82.3), (80.0), 79.8, 53.7, (53.4), 52.5, (52.1), 41.6, (41.2), 35.3, (34.5), 28.9, (28.8), 28.34, 28.0, (27.9). HRMS (ESI$^+$) calcd *m/z* for $C_{28}H_{27}N_2O_5$ $[M+H]^+$, 471.1914 found 471.1912.

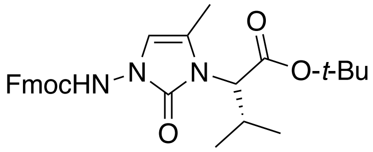

40

Fmoc-(5-Me)Nai-Val-O*t*-Bu (40)

2,6-Lutidine (0.85 mL, 7.3 mmol, 1.15 equiv.) was added slowly to a stirred suspension of Fmoc-aza-Gly-Val-O*t*-Bu (**41**, 2.87 g, 6.3 mmol, 1.0 equiv., prepared according to the protocol described below) and *N*-bromosuccinimide (NBS, 1.3 g, 7.3 mmol, 1.15 equiv.) in 100 mL of anhydrous CH_2Cl_2 at 0°C, and stirred for 10 min, when the color of the reaction mixture changed from sunset orange to yellow orange. Water (35 mL) was added to the reaction mixture, followed by 2 M HCl (16 mL). The phases were separated. The organic layer was washed with a solution of sodium thiosulfate pentahydrate (1.0 g) in water (50 mL), saturated aqueous $NaHCO_3$ (50 mL), and water (50 mL), dried over anhydrous $MgSO_4$, filtered, and evaporated to afford Fmoc-azoGly-Val-O*t*-Bu, which was dissolved in 100 mL of anhydrous DCM. The azopeptide solution was treated with propionaldehyde (1.8 mL, 25.3 mmol, 4.0 equiv.) and L-proline (200 mg, 1.1 mmol, 0.3 equiv.). After stirring overnight at room temperature, the solution became colorless. The volatiles were evaporated to yield a yellow residue, which was purified by column chromatography on silica gel (50% EtOAc in hexanes) to obtain a mixture of the aldehyde and aminol: Rf = 0.51 and 0.57 (30% EtOAc in hexanes). The mixture was dissolved in $CHCl_3$ (280 mL), treated with *p*-TsOH·H_2O (240 mg, 1.26 mmol, 0.2 equiv.), stirred for 2h, transferred into a separatory funnel and washed with saturated sodium bicarbonate (40 mL), and brine. The organic layer was dried over Na_2SO_4, filtered, and evaporated to a residue, which was purified by column chromatography (50% EtOAc in hexanes). Evaporation of the collected fractions gave Fmoc-(5-Me)Nai-Val-O*t*-Bu (**40**, 2.49 g, 80 % overall yield from Fmoc-azaGly-Val-O*t*-Bu **41**) as a white foam: R_f = 0.84 (30% EtOAc in hexanes); $[\alpha]_D^{23}$ –0.86 (*c* 0.83, $CHCl_3$); ^1H NMR (400 MHz, $CDCl_3$) δ 8.09 (s, 1 H), 7.72 (d, *J* = 7.6 Hz, 2H), 7.59 (m, 2H), 7.38 (t, *J* = 7.4 Hz, 2H), 7.28 (t, *J* = 9 Hz, 2H), 6.30 (s, 1H), 4.55 (d, *J*

= 8.8 Hz, 1H), 4.35 (s, 2H), 4.21 (s, 1H), 2.33 – 2.14 (m, 1H), 1.99 (s, 3H), 1.47 (s, 9H), 0.99 (d, *J* = 6.7 Hz, 3H), 0.87 (d, *J* = 6.8 Hz, 3H); ^{13}C NMR (101 MHz, $CDCl_3$) δ 169.7, 155.8, 152.5, 143.7, 141.4, 127.9, 127.3, 125.4, 120.1, 104.2, 82.5, 68.3, 61.4, 60.6, 47.1, 31.8, 28.1, 19.3, 18.8, 9.4; HRMS (ESI+) calcd m/z for $C_{28}H_{33}N_3O_5Na$ [M+Na]+ 514.2312, found: 514.2297.

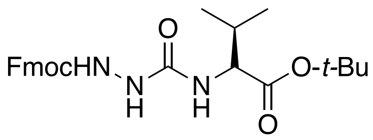

41

Fmoc-azaGly-Val-O*t*-Bu (41)

A solution of *N,N'*-disuccinimidyl carbonate (DSC, 2.7 g, 10.6 mmol, 1.1 equiv.) in anhydrous THF (100 mL) under argon was cooled to 0°C and treated with Fmoc-hydrazide (2.7 g, 9.6 mmol). The ice bath was removed. The reaction mixture warmed to room temperature. After stirring for 1 h, the mixture was cooled to 0°C and treated with L-valine *tert*-butyl ester hydrochloride (2.2 g, 10.6 mmol, 1.1 equiv.) and DIEA (3.7 mL, 21.1 mmol, 2.2 equiv.). The ice bath was removed. The reaction warmed to room temperature with stirring overnight. The volatiles were evaporated. The residue was partitioned between EtOAc (50 mL) and water (50 mL). The aqueous layer was extracted with EtOAc (3 x 25 mL). The organic layers were combined, washed brine, dried with $MgSO_4$, filtered and evaporated to a residue, which was purified by silica gel chromatography eluting with 50% EtOAc in hexane as eluent. Evaporation of the collected fractions gave *N*-(Fmoc)aza-glycinyl valine *tert*-butyl ester (**41**, 2.9 g, 66%) as white foam: R_f= 0.57 (50% EtOAc in hexanes); $[\alpha]_D^{23}$ 0.86 (*c* 0.83, $CHCl_3$); ^1H NMR (400 MHz, chloroform-*d*) δ 7.75 (dt, *J* = 7.6, 0.9 Hz, 2H), 7.63 – 7.53 (m, 2H), 7.39 (tt, *J* = 7.6, 2.0 Hz, 2H), 7.29 (tt, *J* = 7.5, 1.4 Hz, 2H), 4.47 – 4.39 (m, 1H), 4.35 (dd, *J* = 8.5, 4.5 Hz, 1H), 4.23 (t, *J* = 7.1 Hz, 1H), 3.76 – 3.60 (m, 1H), 2.15 (m, 1H), 1.44 (s, 9H), 0.94 (d, *J* = 6.9 Hz, 3H), 0.87 (d, *J* = 6.9 Hz, 3H); $^{13}C[^1H]$ NMR (101 MHz, $CDCl_3$) δ 171.9, 158.1, 156.9, 143.6, 141.4, 128.0, 127.3, 125.3, 120.2, 82.3, 62.9, 58.3, 47.0, 31.7, 28.2, 19.0, 17.7; HRMS (ESI^+) calcd m/z for $C_{25}H_{31}N_3O_5Na$ $[M+Na]^+$ 476.21559, found: 476.21482.

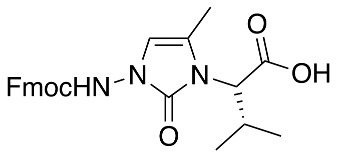

45

Fmoc-(5-Me)Nai-Val-OH (45)

A solution of dipeptide **40** (718 mg, 1.46 mmol) in CH_2Cl_2 (10 mL) was cooled to 0°C, and treated with trifluoracetic acid (TFA, 10 mL). The solution was warmed to room temperature and was stirred until all starting material was observed to be consumed by TLC. The solution was concentrated *in vacuo* and dissolved in CH_2Cl_2 and evaporated three times to afford acid **45**, which was used in the next step without further purification.

NMR Spectra

16, DMSO-*d6*, ^{1}H (700 MHz)

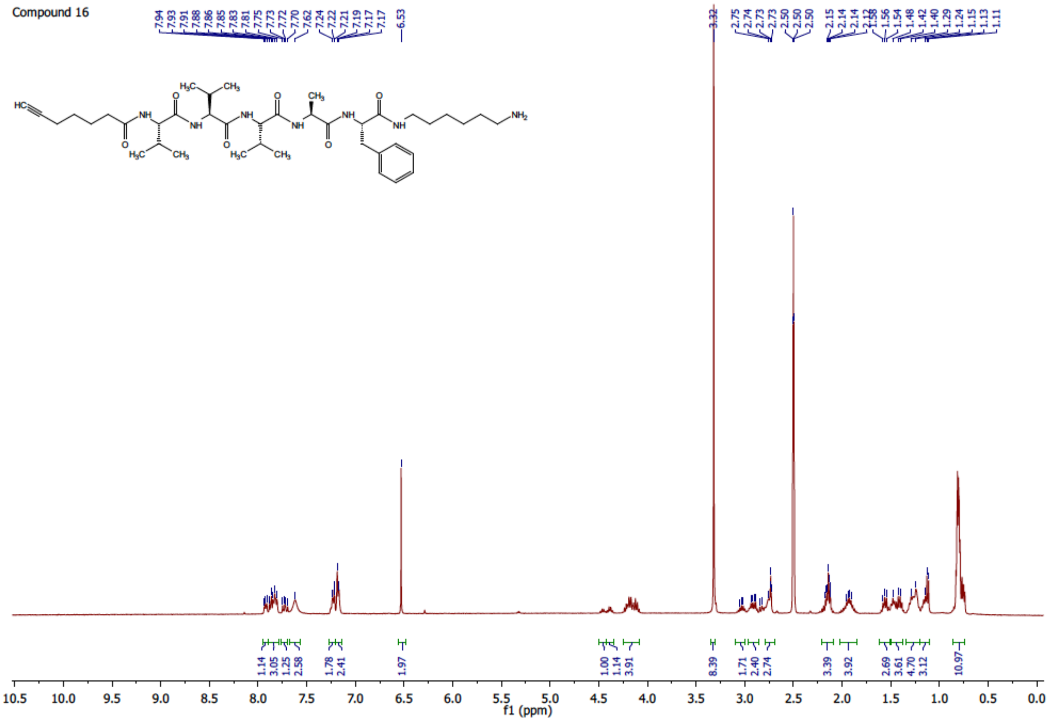

18, DMSO-*d6*, ^{1}H (700 MHz)

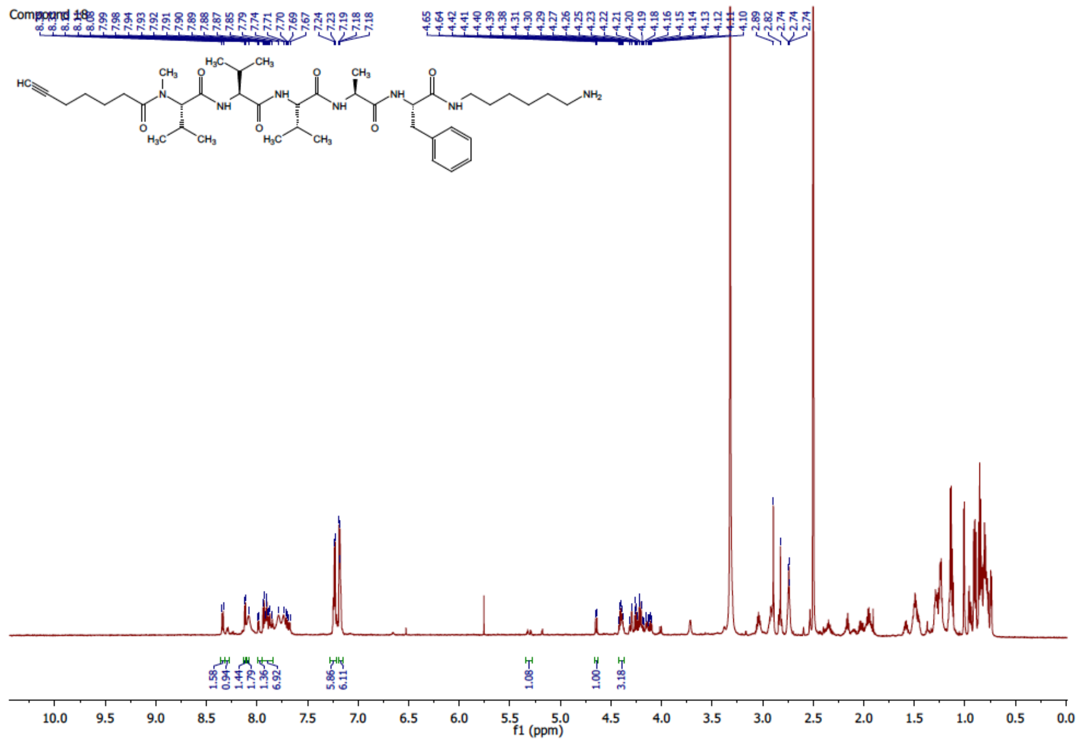

38b, $CDCl_3$, ^{1}H (500 MHz), ^{13}C (126 MHz)

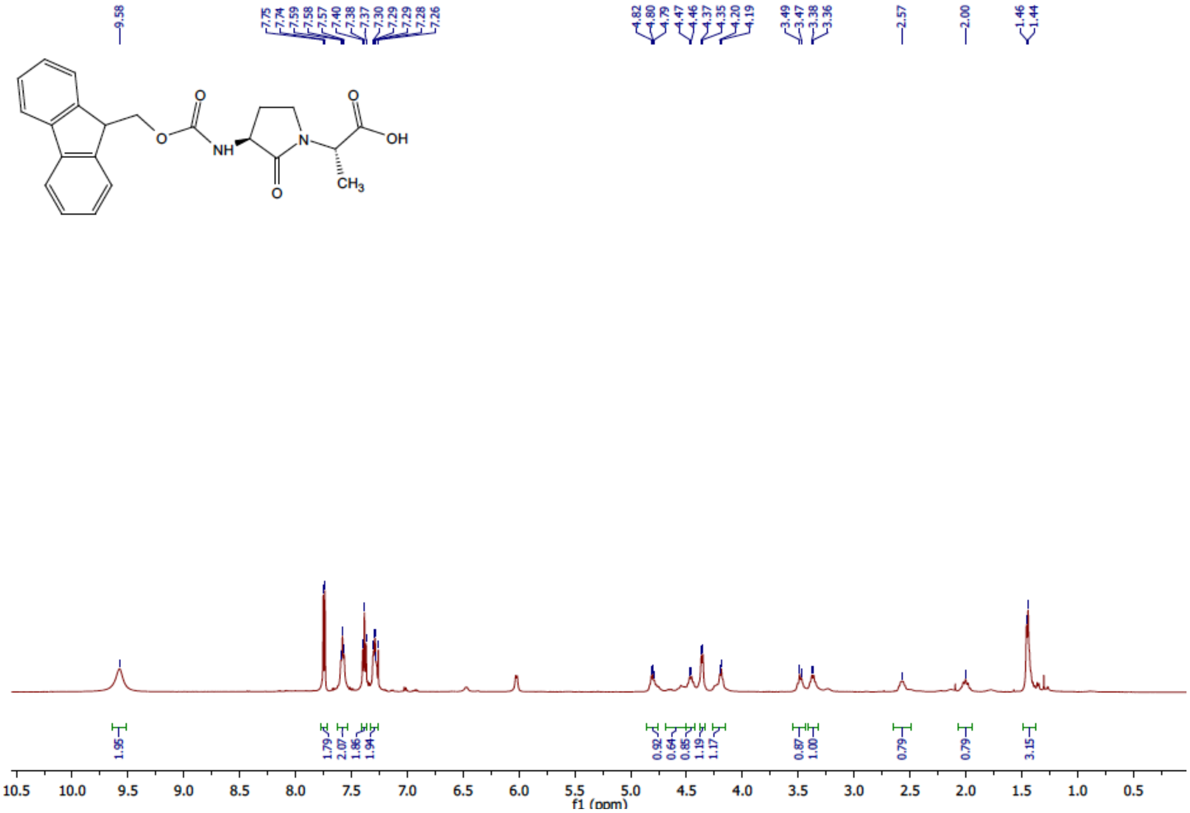

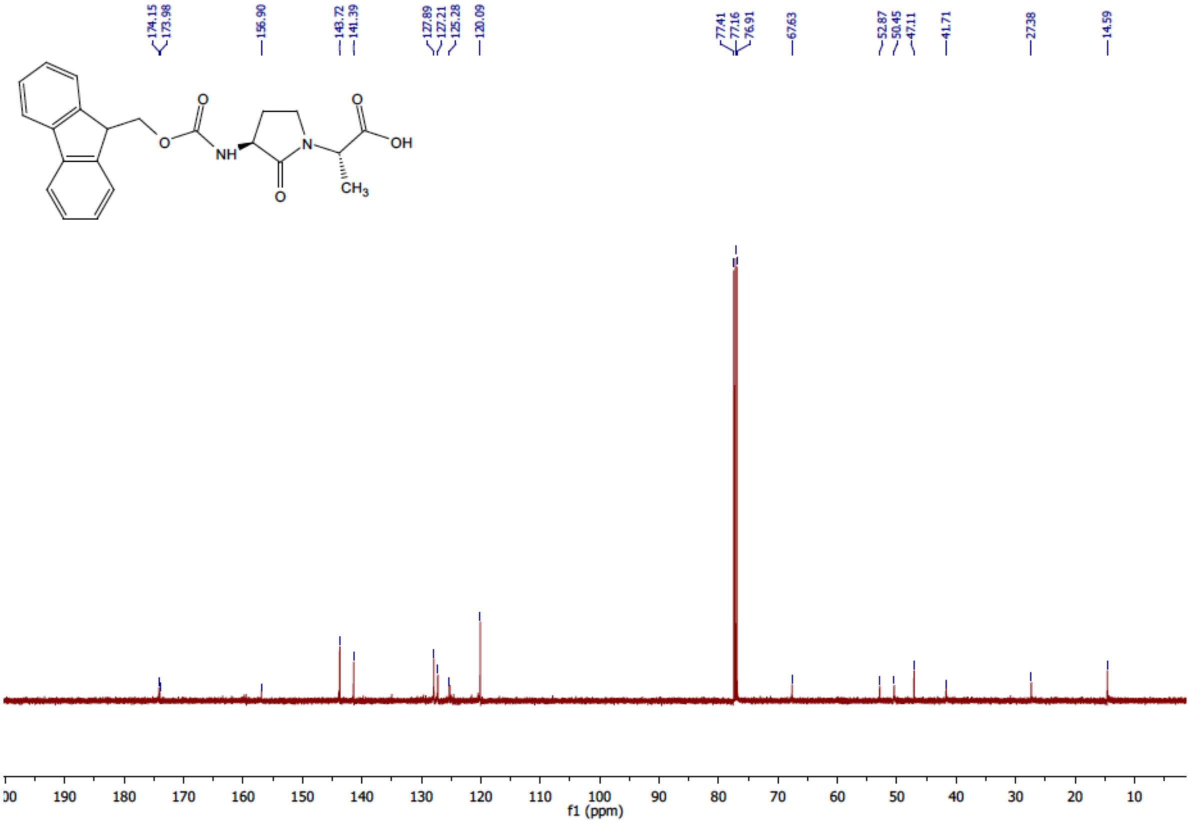

38c, $CDCl_3$, ^{1}H (500 MHz), ^{13}C (126 MHz)

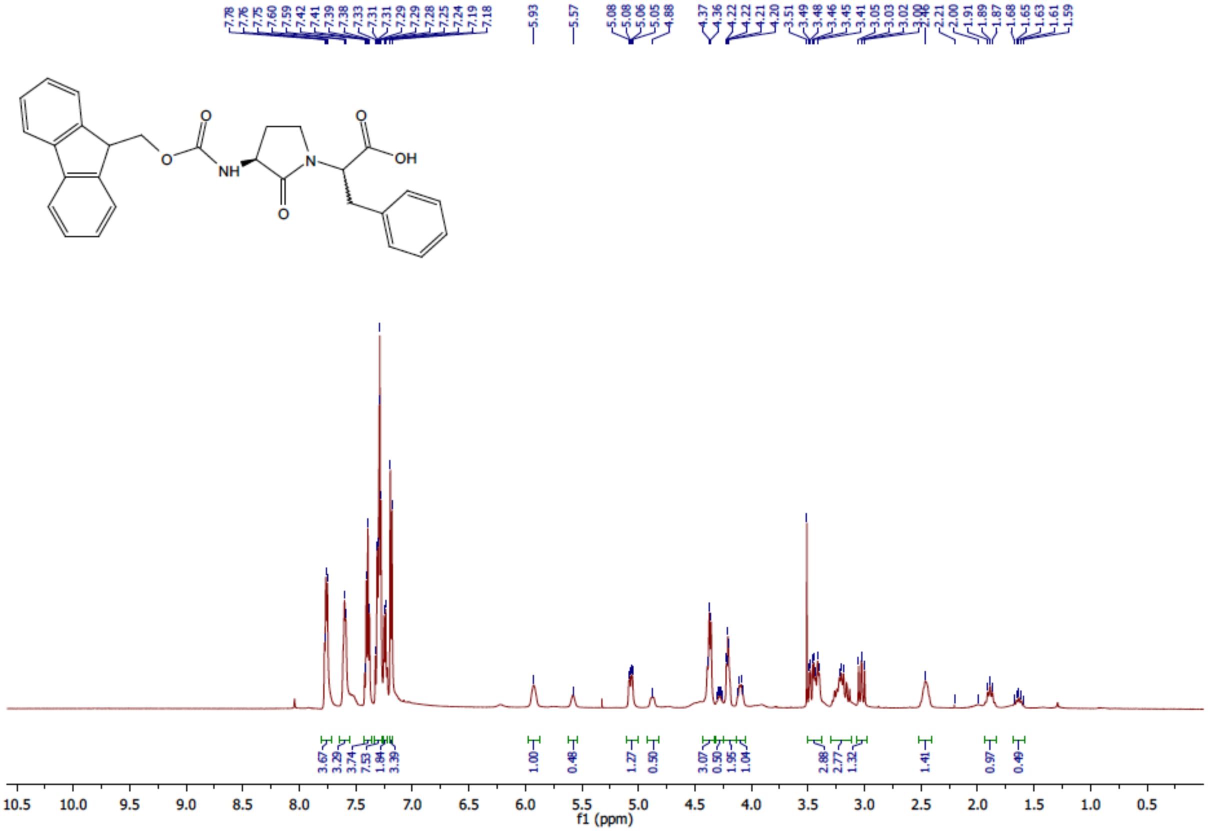

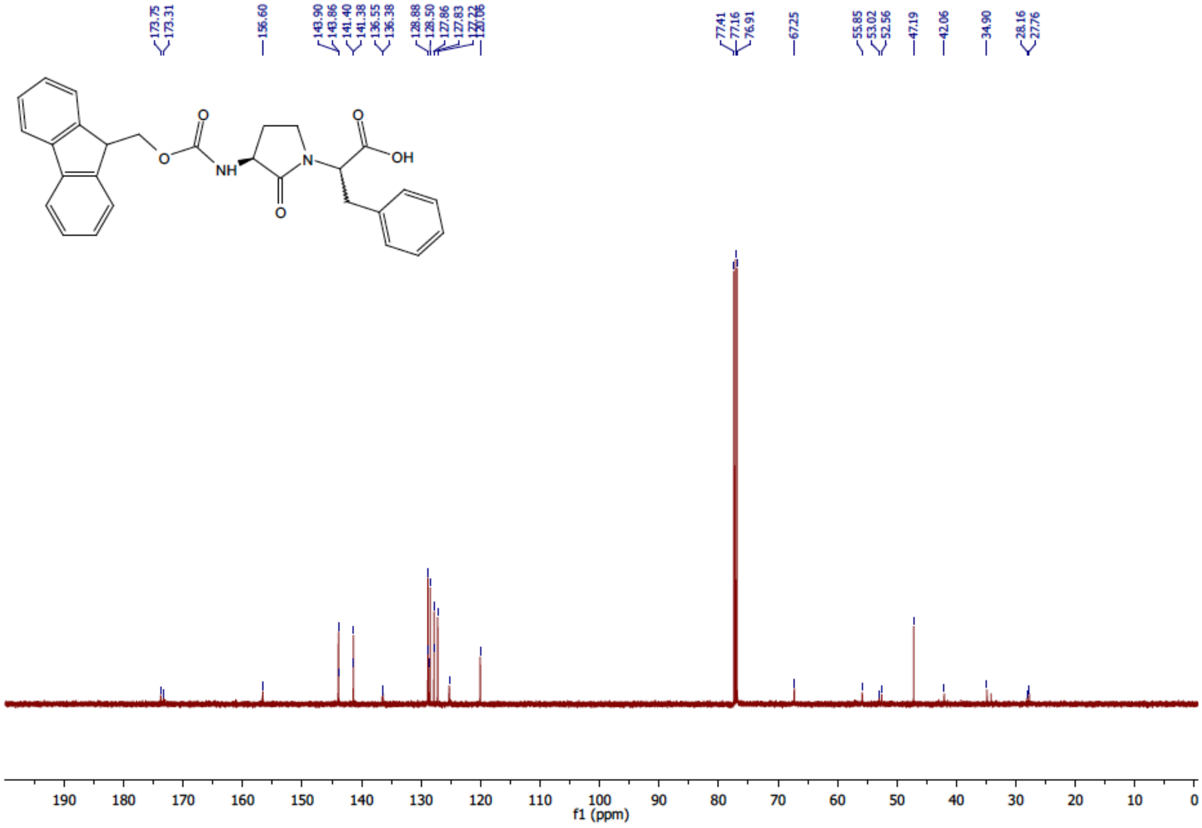

39b, $CDCl_3$, ^1H (500 MHz), ^{13}C (126 MHz)

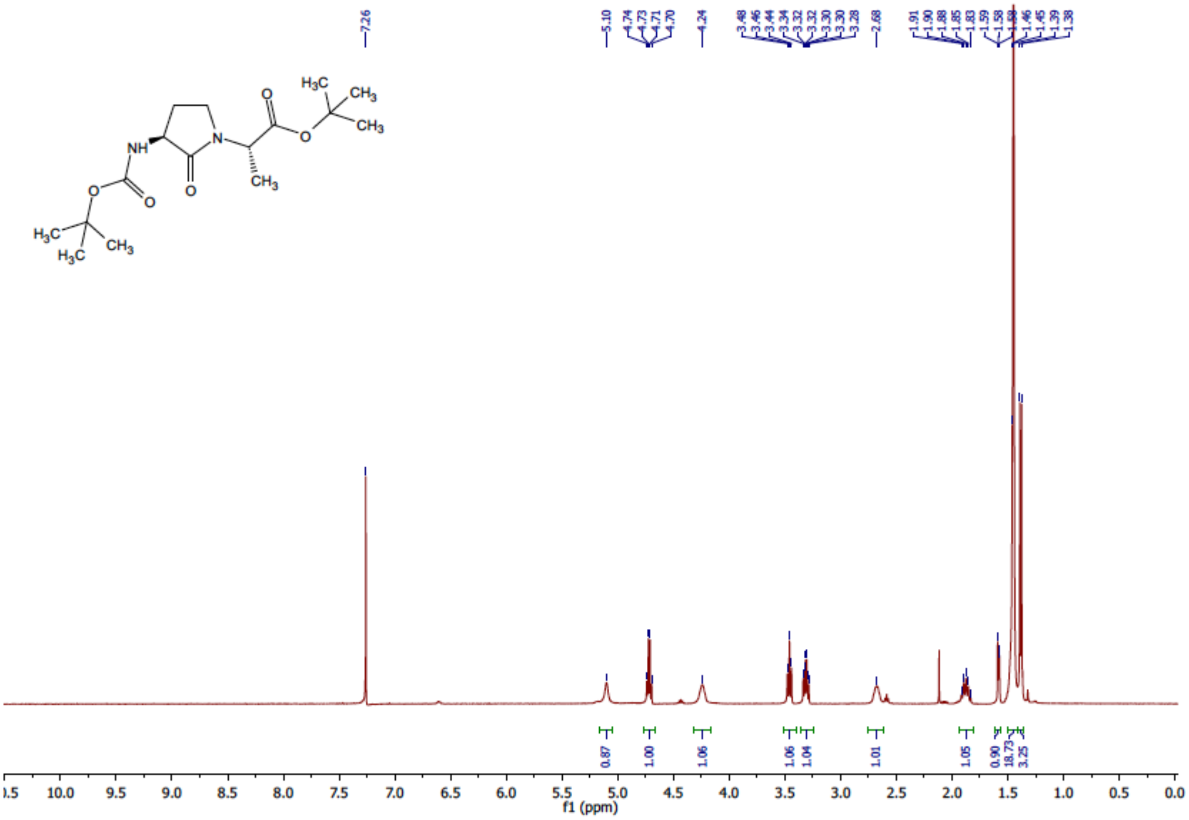

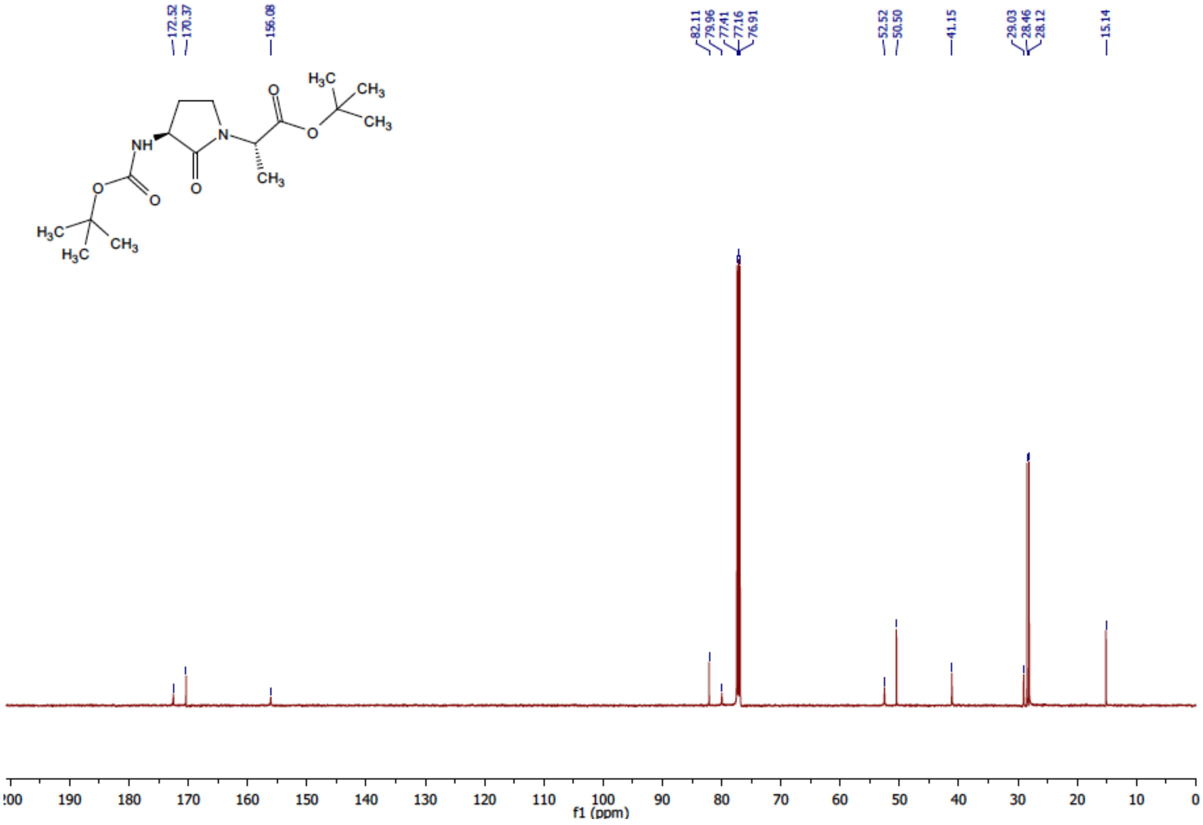

39c, $CDCl_3$, ^{1}H (500 MHz), ^{13}C (126 MHz)

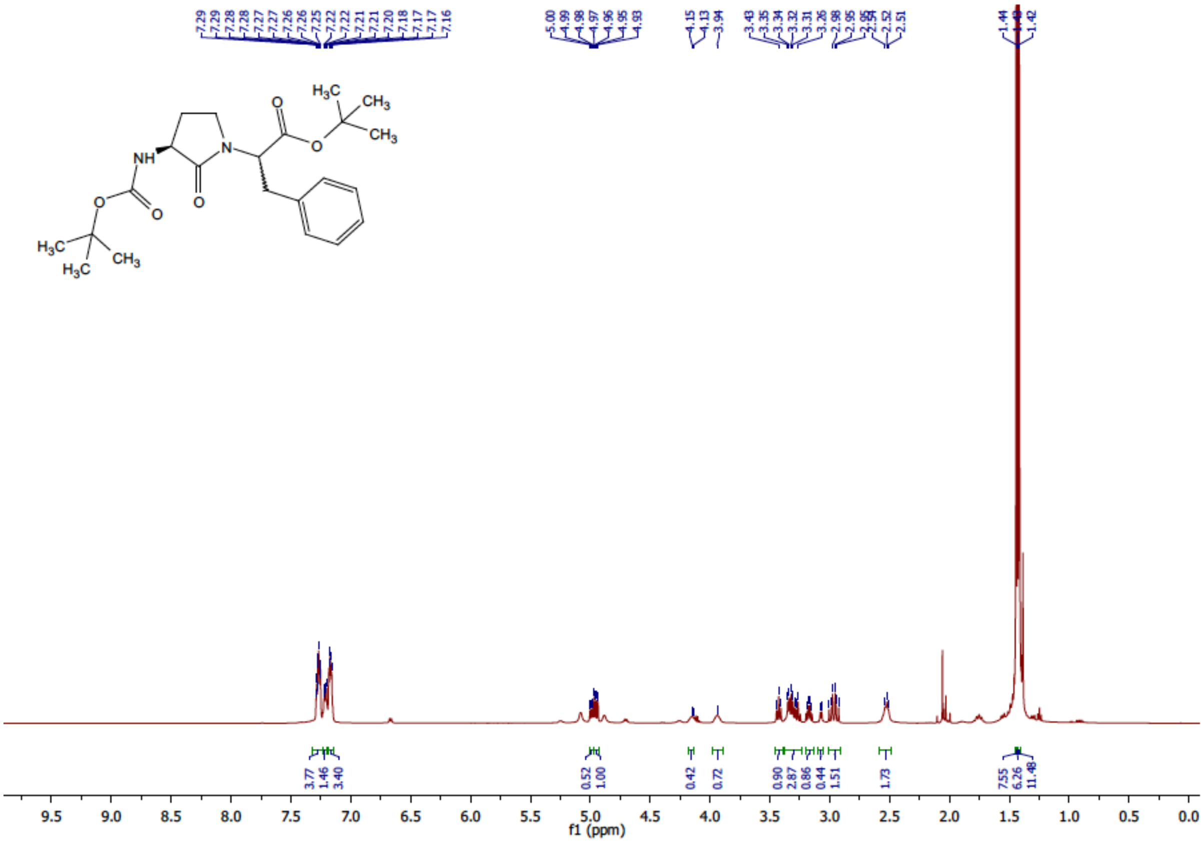

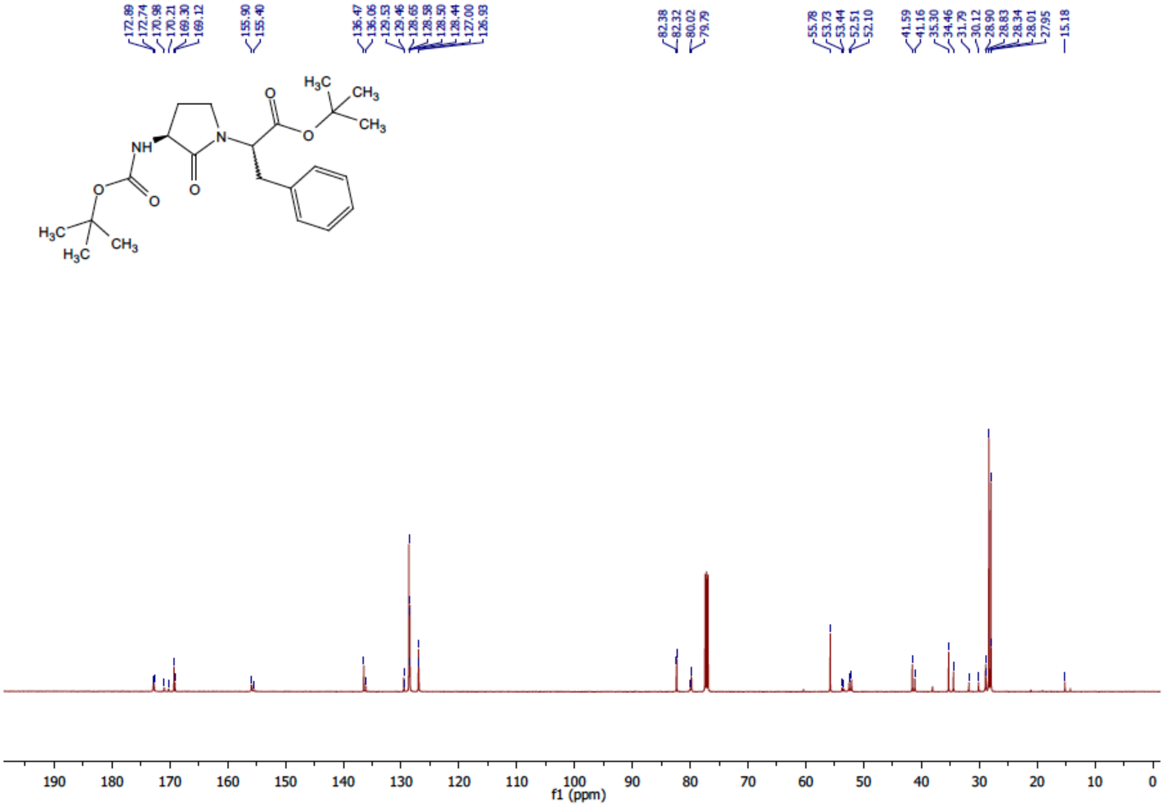

40, $CDCl_3$, ^{1}H (400 MHz), ^{13}C (101 MHz)

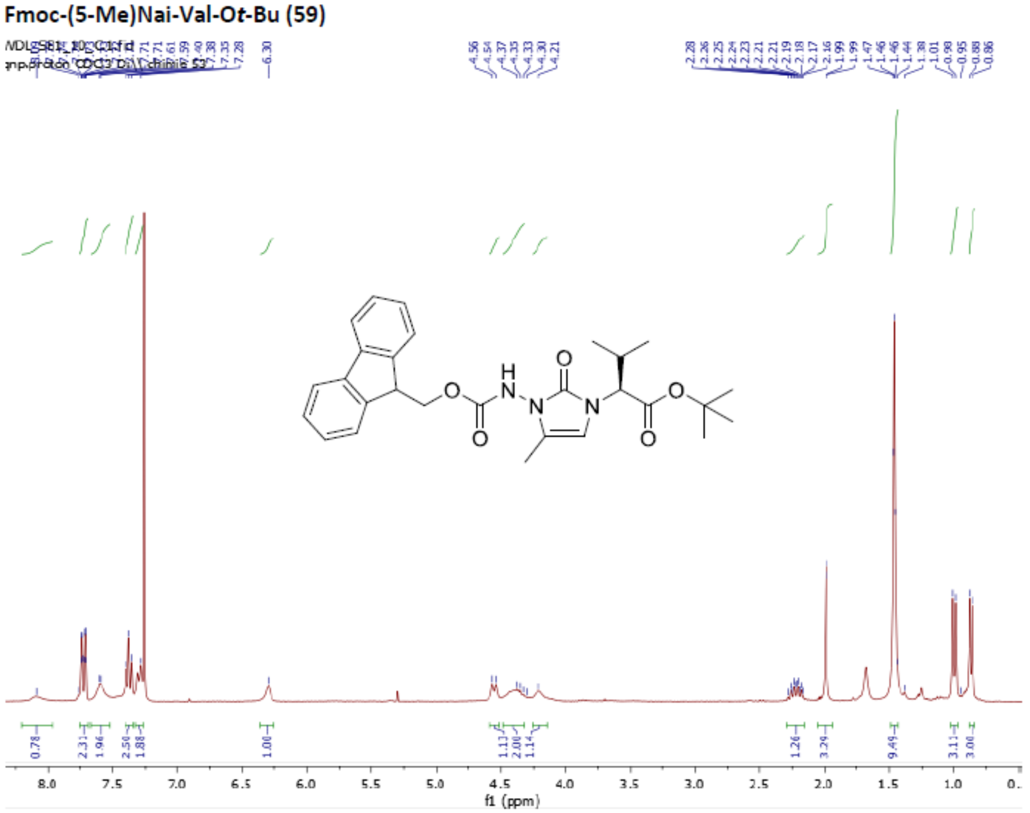

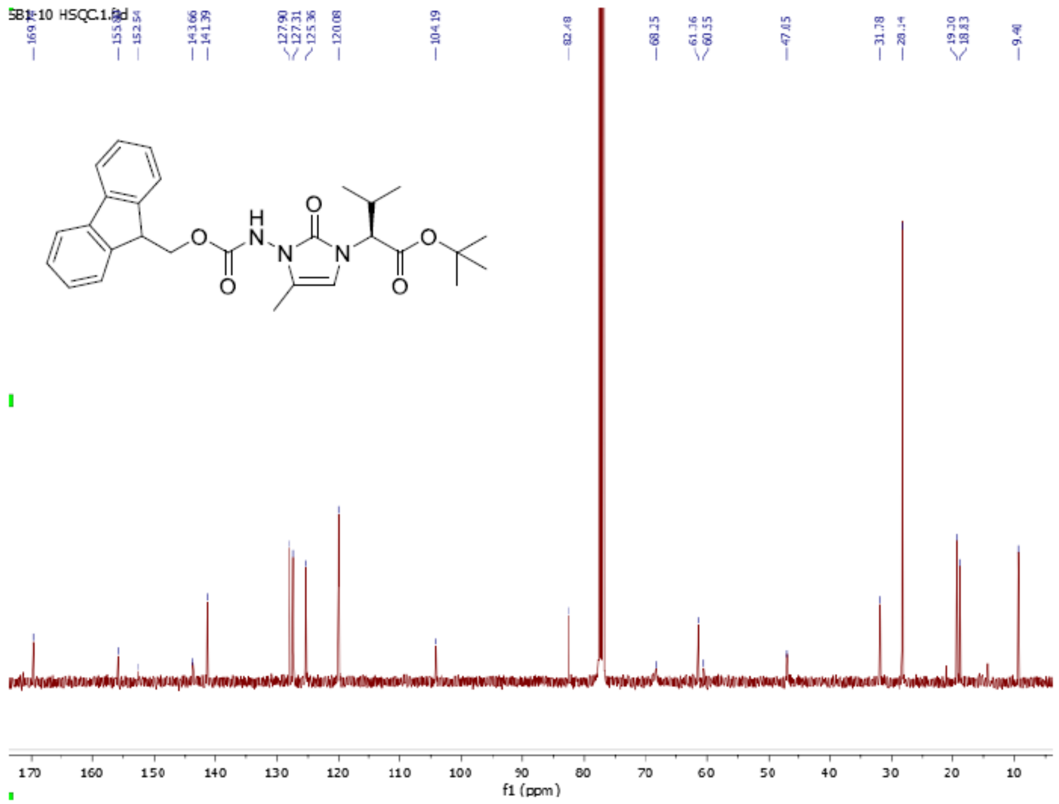

41, $CDCl_3$, ^{1}H (400 MHz), ^{13}C (101 MHz)

Fmoc-azaGly-Val-O*t*-Bu (56)

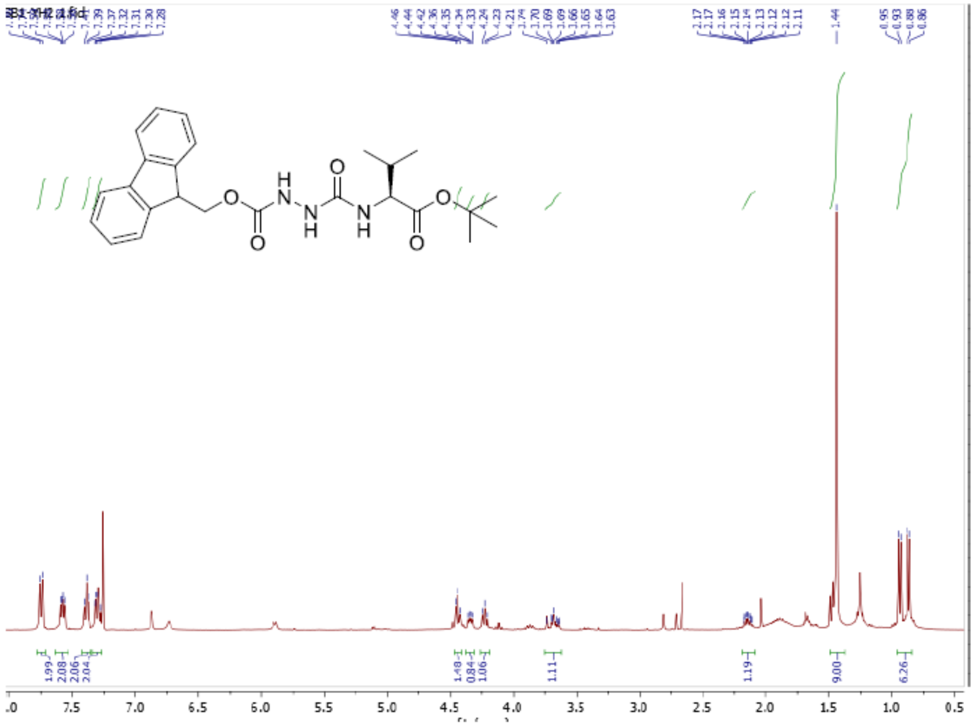

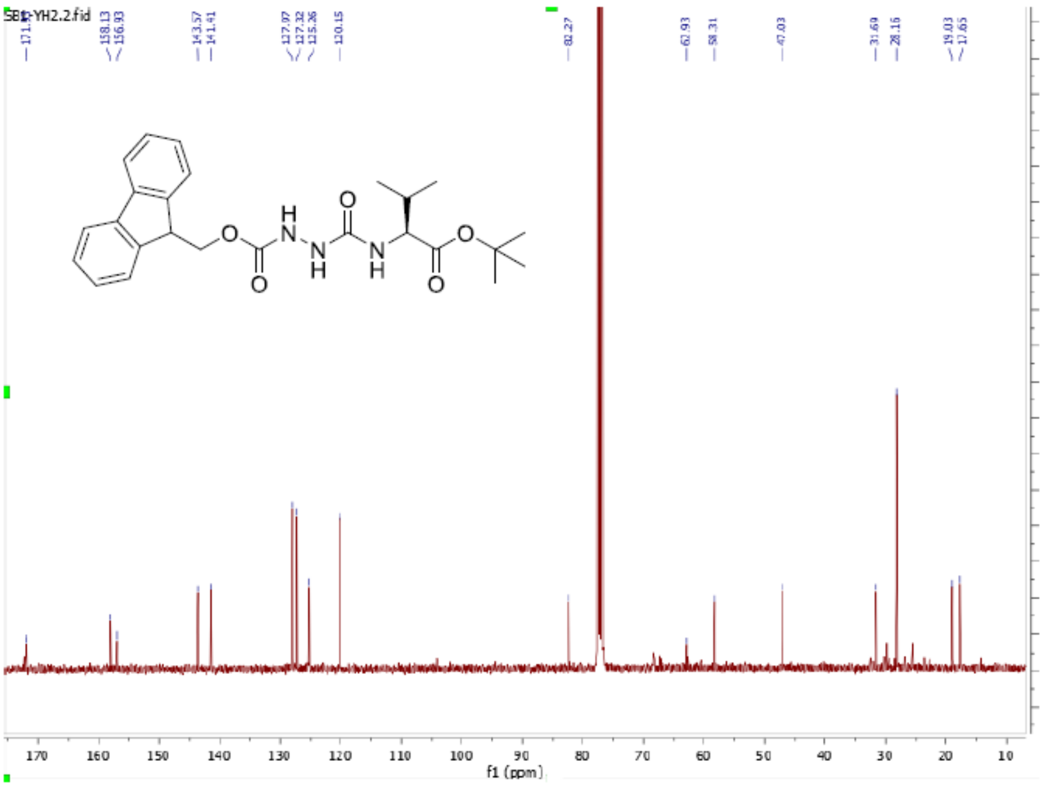

Ascertainment of purity by HPLC

Method A: Analytical HPLC, 10 to 90% methanol [0.1% formic acid (FA)] in water (0.1% FA) over 14 min, flow rate of 0.5 mL/min on a Gemini reverse-phase column from Phenomenex (4.6 mm × 50 mm, 5 µm, C18).

Method B: Analytical HPLC, 30 to 95% methanol [0.1% formic acid (FA)] in water (0.1% FA) over 14 min, flow rate of 0.5 mL/min on a Gemini reverse-phase column from Phenomenex (4.6 mm × 50 mm, 5 µm, C18).

Method C: Analytical HPLC, 50 to 90% methanol [0.1% formic acid (FA)] in water (0.1% FA) over 14 min, flow rate of 0.5 mL/min on a Gemini reverse-phase column from Phenomenex (4.6 mm × 50 mm, 5 µm, C18).

Method D: Analytical HPLC, 10 to 90% acetonitrile [0.1% formic acid (FA)] in water (0.1% FA) over 14 min, flow rate of 0.5 mL/min on a Gemini reverse-phase column from Phenomenex (4.6 mm × 50 mm, 5 µm, C18).

Method B, **12**, Purity ≥ 99% (MeOH)

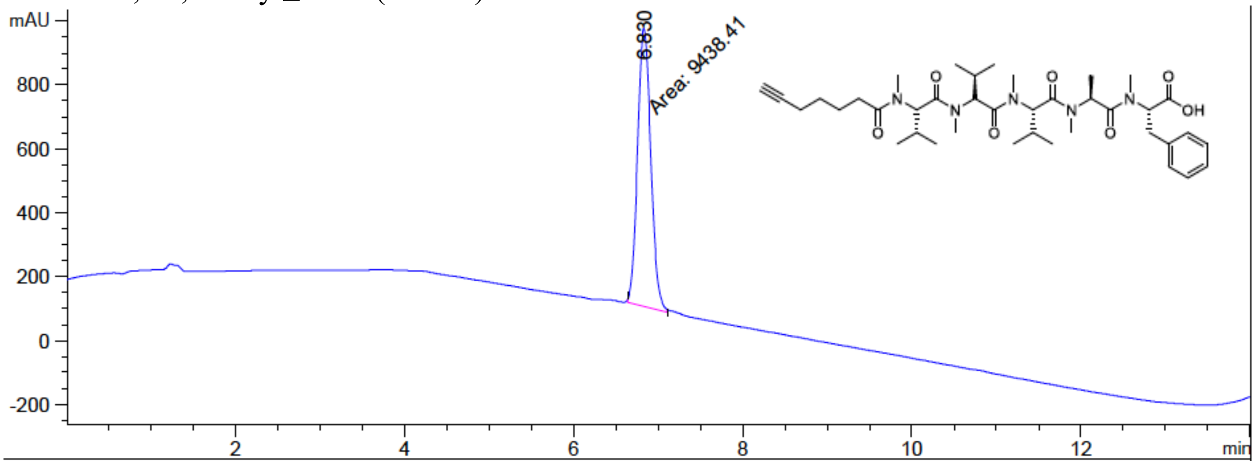

Method D, **12**, Purity ≥ 99% (MeCN)

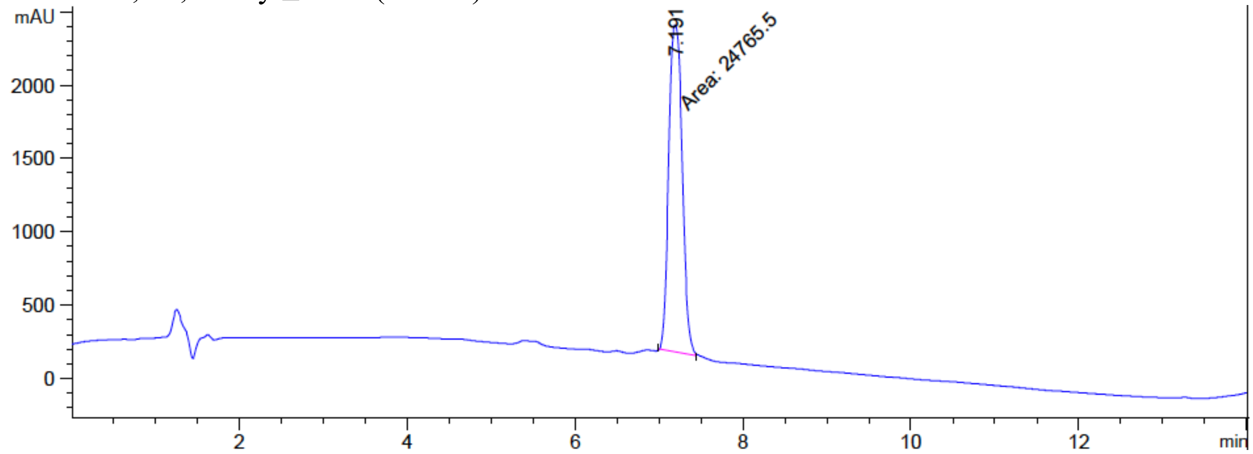

Method B, **15**, Purity ≥ 99% (MeOH)

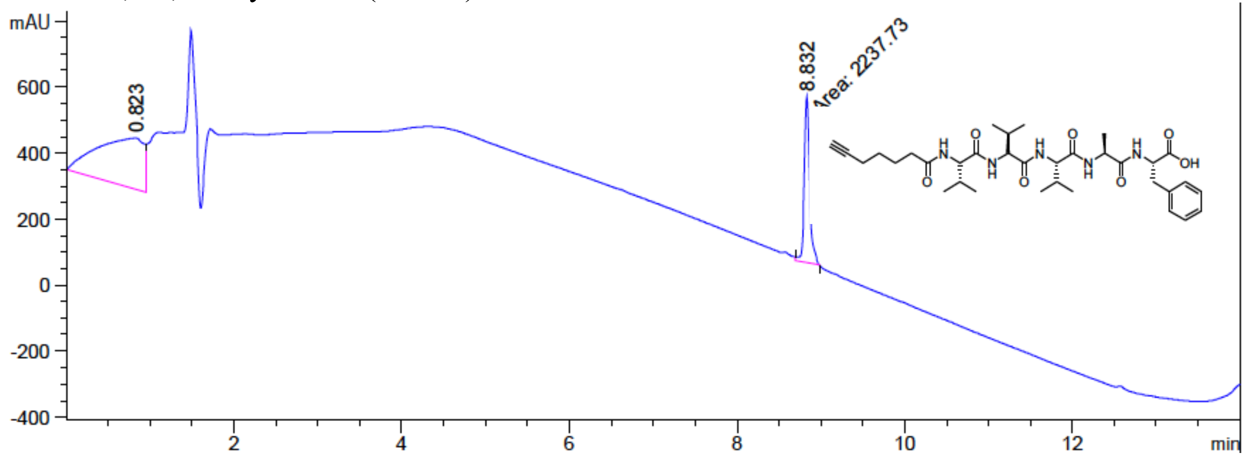

Method D, **15**, Purity ≥ 99% (MeCN)

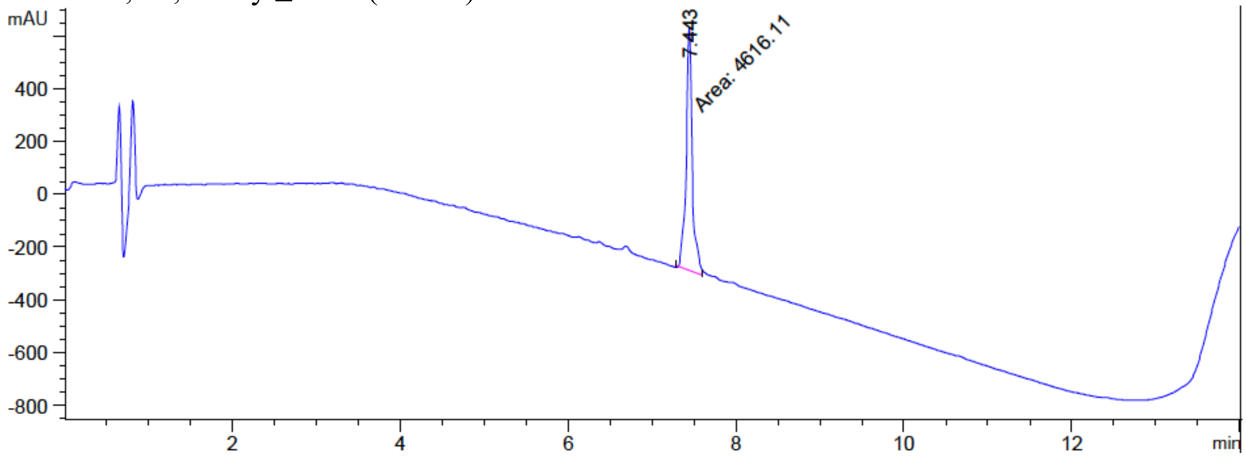

Method B, **16**, Purity ≥ 99% (MeOH)

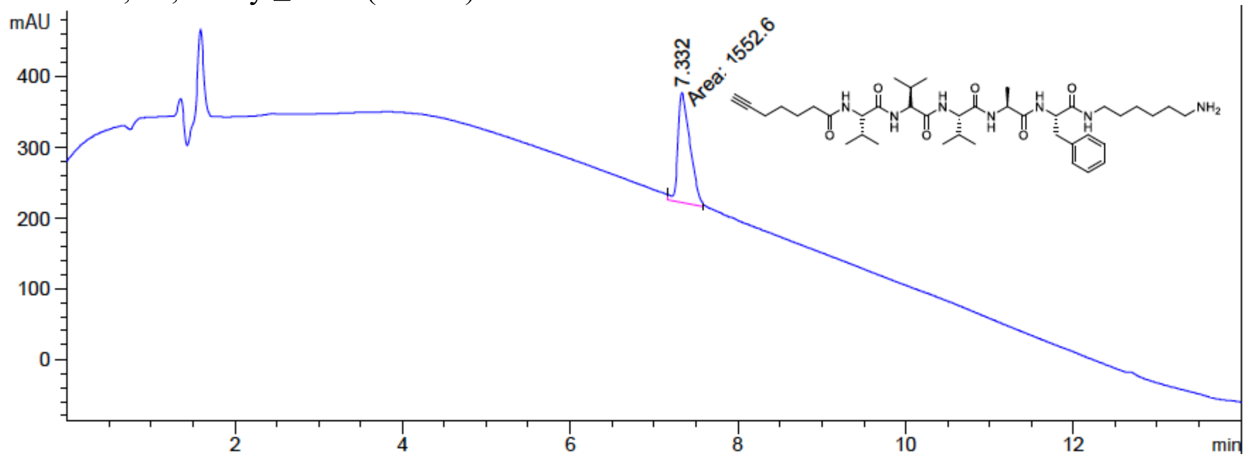

Method D, **16**, Purity ≥ 99% (MeCN)

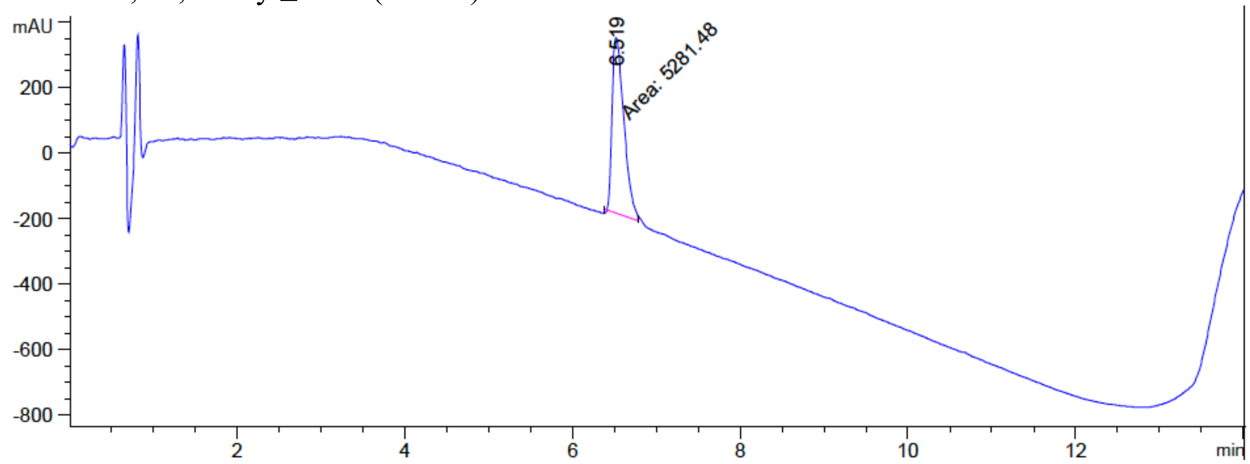

Method B, **17**, Purity ≥ 99% (MeOH)

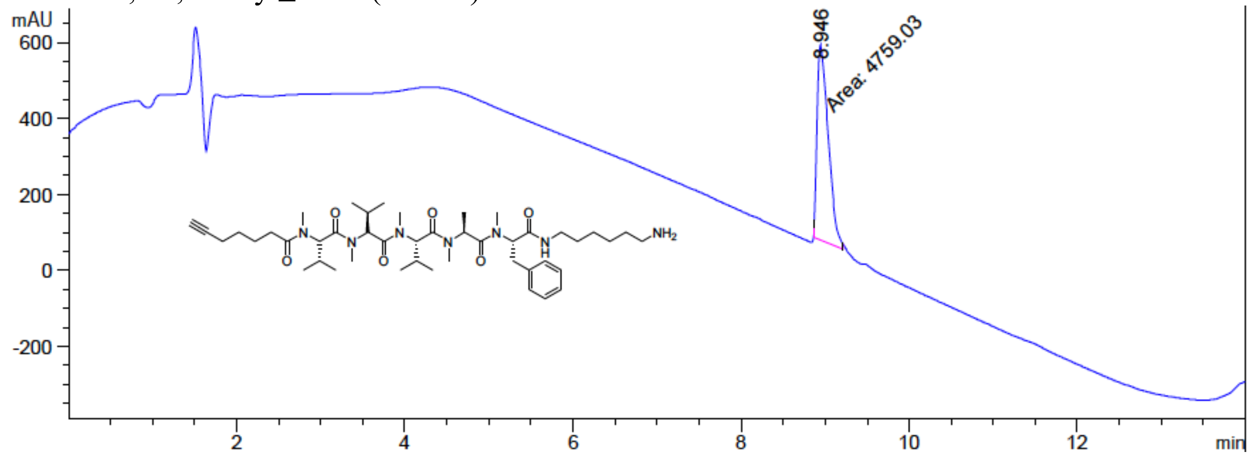

Method D, **17**, Purity ≥ 99% (MeCN)

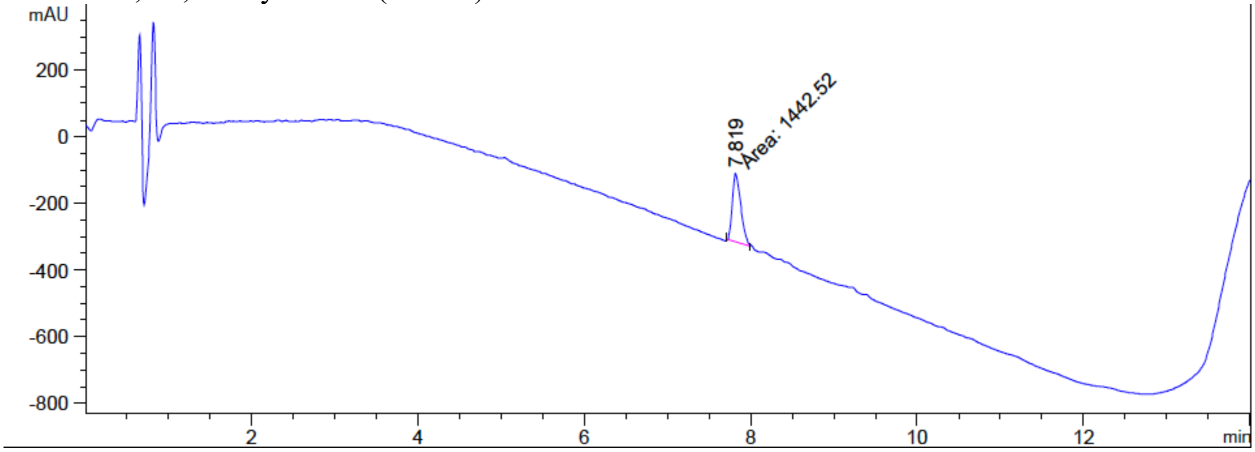

Method B, **18**, Purity ≥ 99% (MeOH)

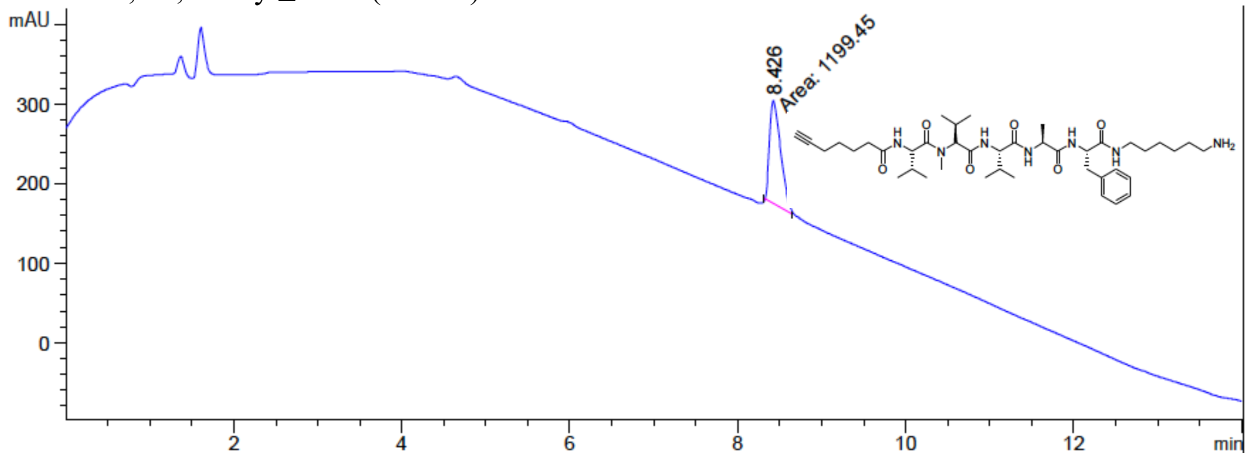

Method D, **18**, Purity ≥ 97% (MeCN)

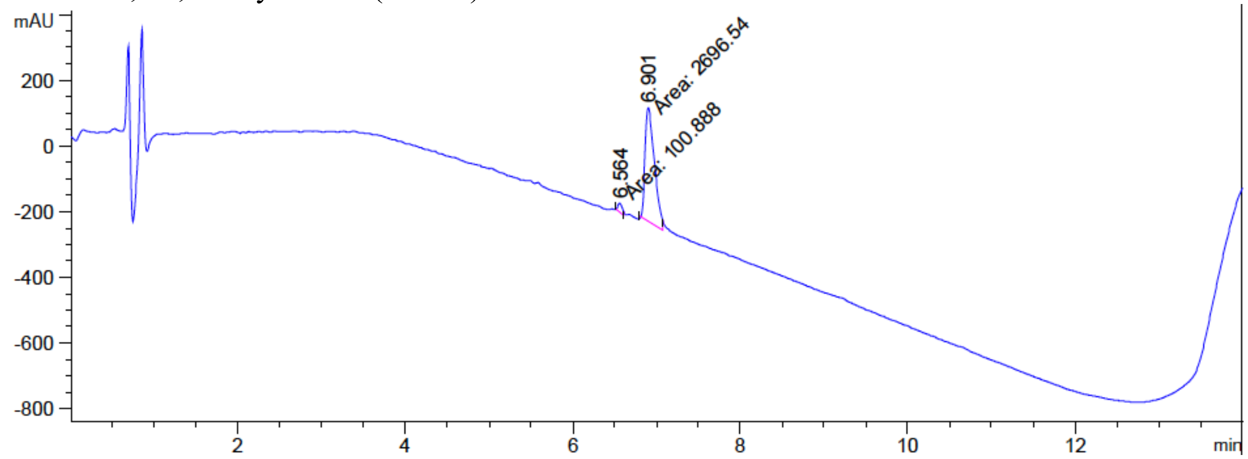

Method B, **19**, Purity ≥ 99% (MeOH)

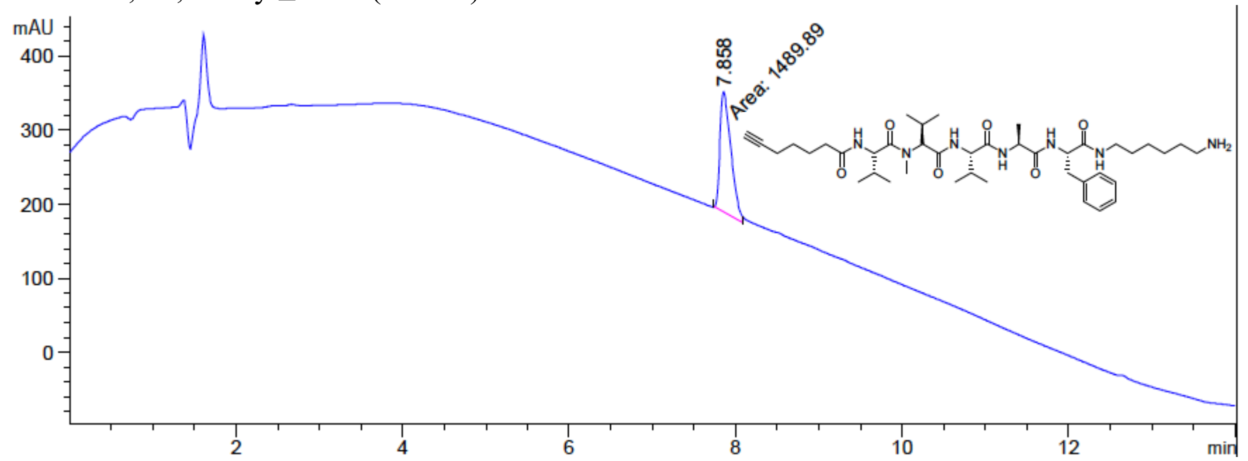

Method D, **19**, Purity ≥ 95% (MeCN)

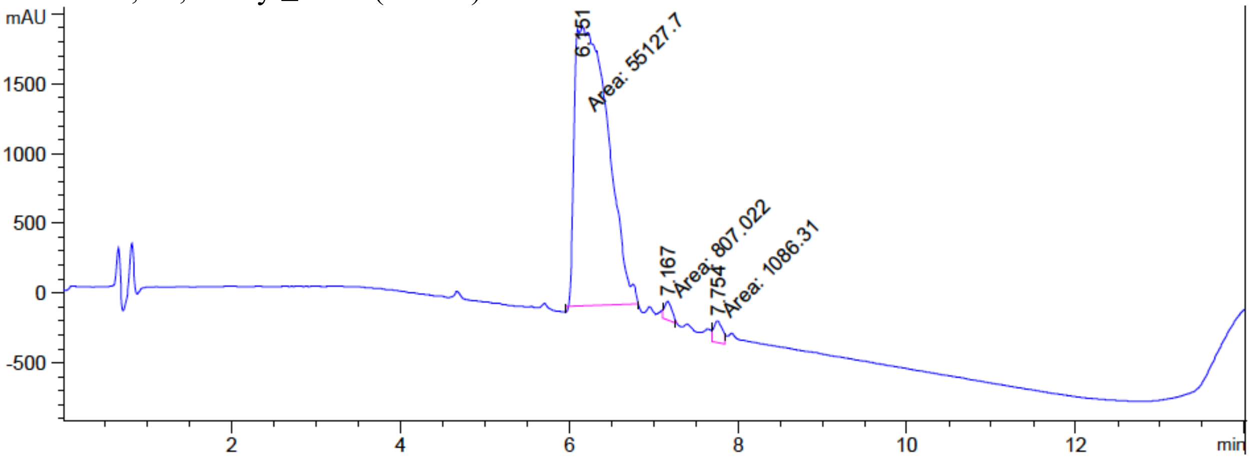

Method B, **20**, Purity ≥ 99% (MeOH)

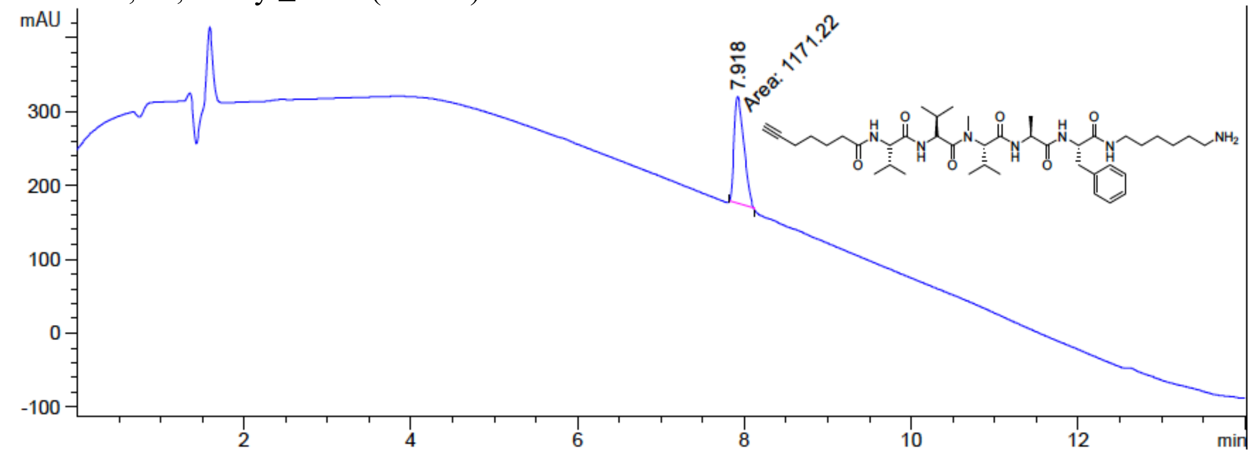

Method D, **20**, Purity ≥ 98% (MeCN)

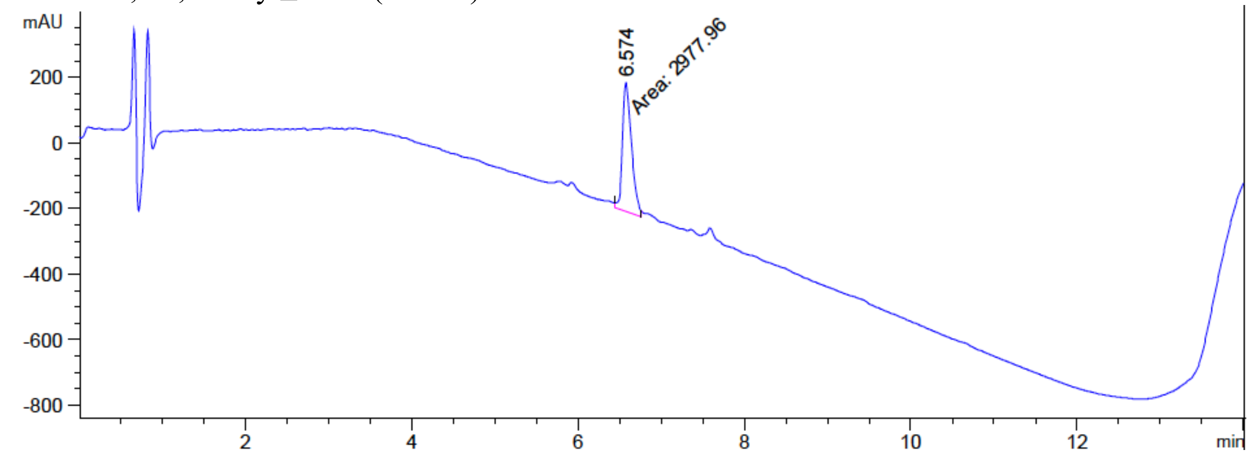

Method B, **21**, Purity ≥ 99% (MeOH)

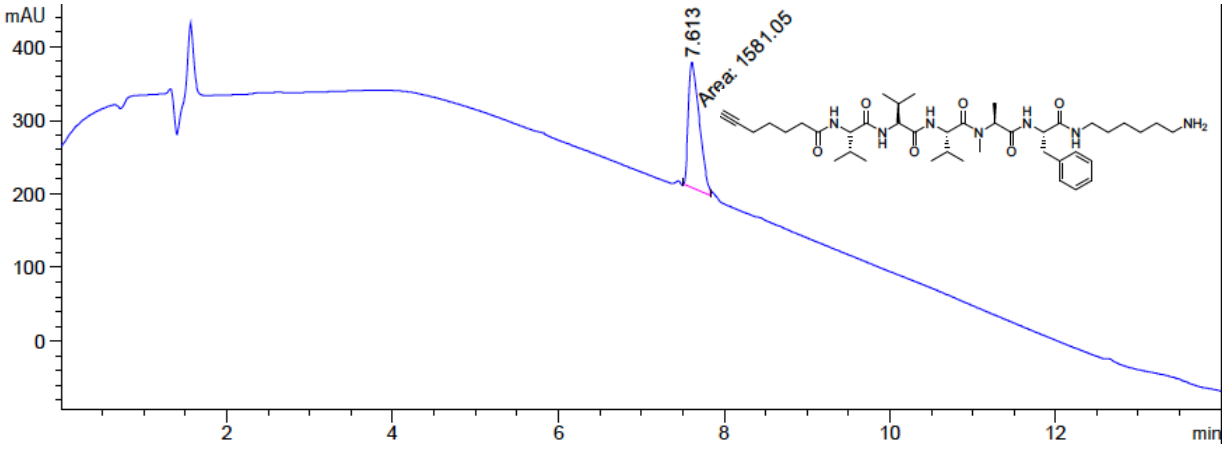

Method D, **21**, Purity ≥ 99% (MeCN)

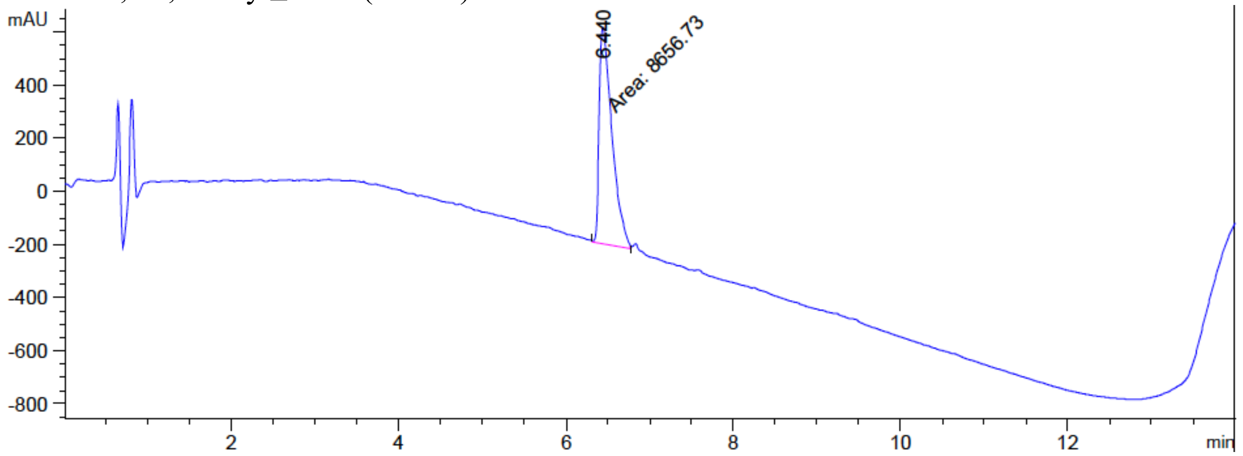

Method B, **22**, Purity ≥ 99% (MeOH)

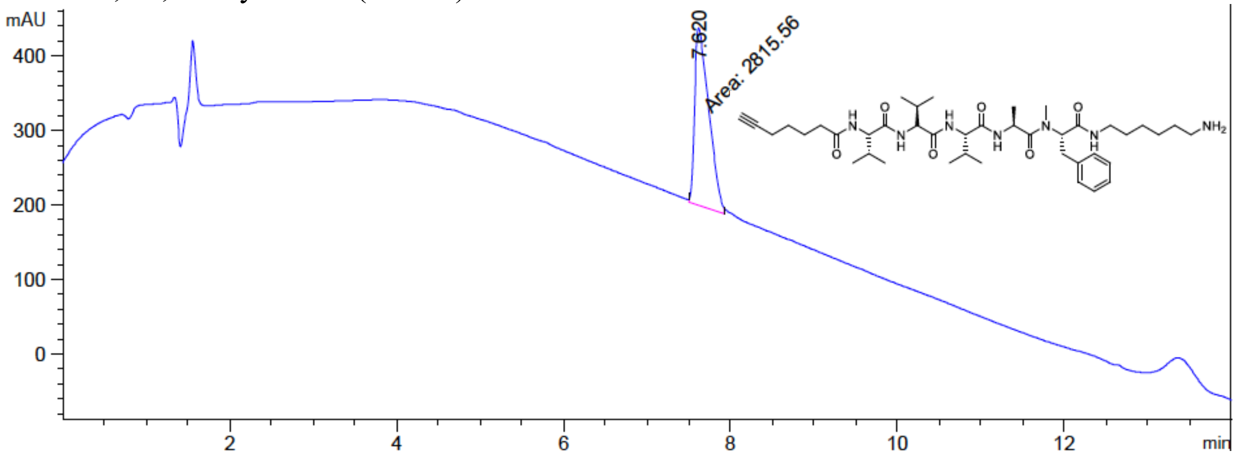

Method D, **22**, Purity ≥ 99% (MeCN)

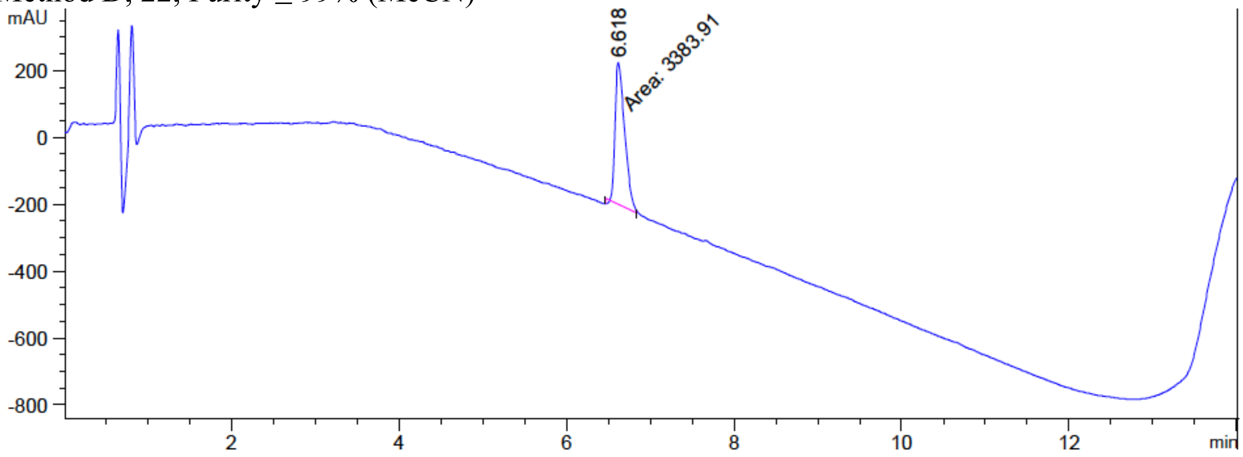

Method C, **23**, Purity ≥ 99% (MeOH)

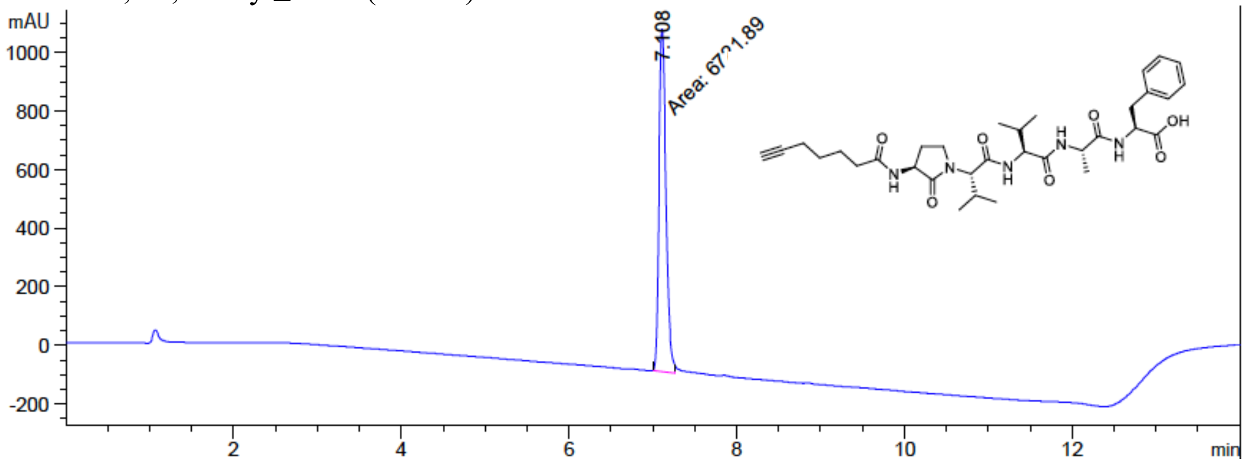

Method D, **23**, Purity ≥ 99% (MeCN)

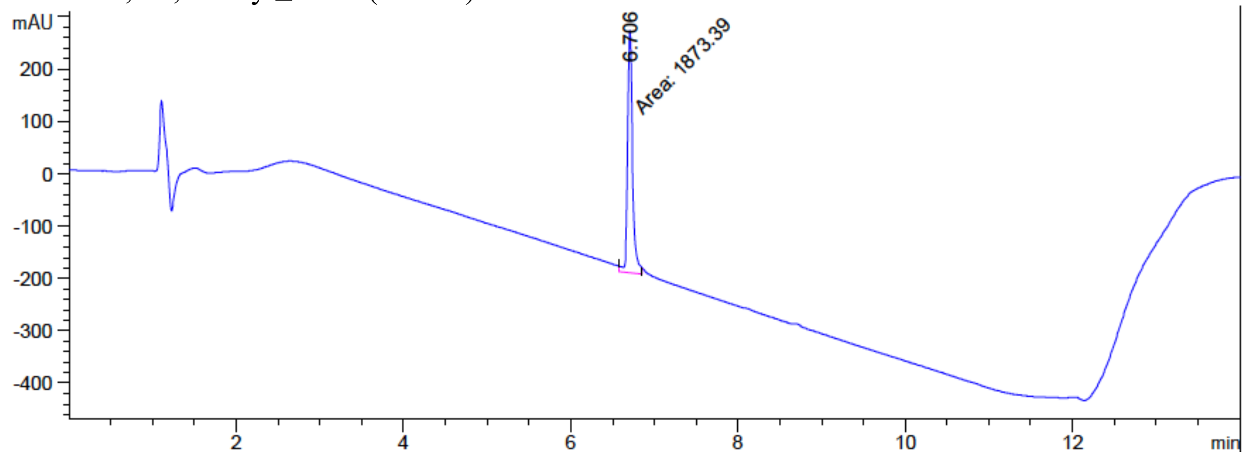

Method C, **24**, Purity ≥ 99% (MeOH)

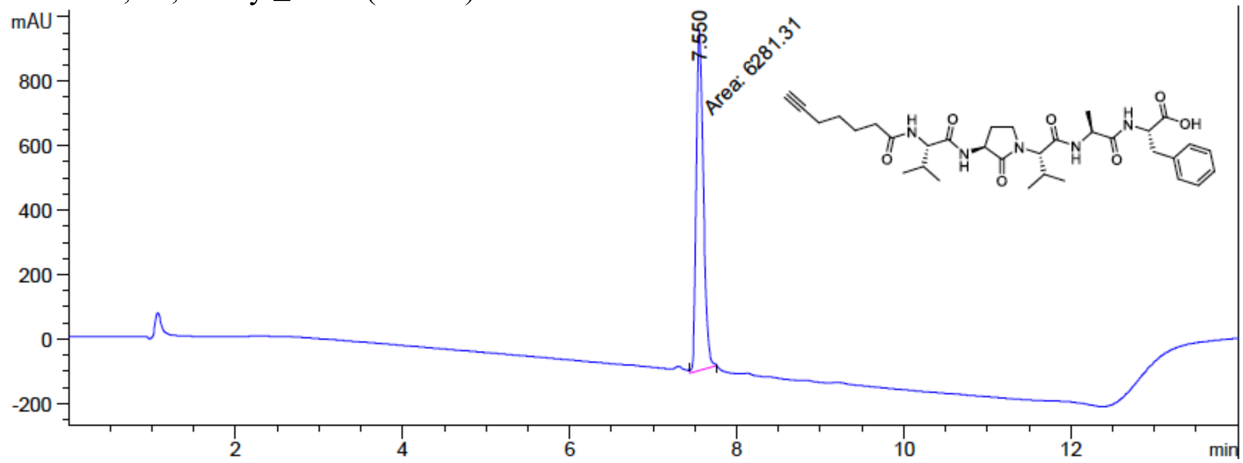

Method D, **24**, Purity ≥ 99% (MeCN)

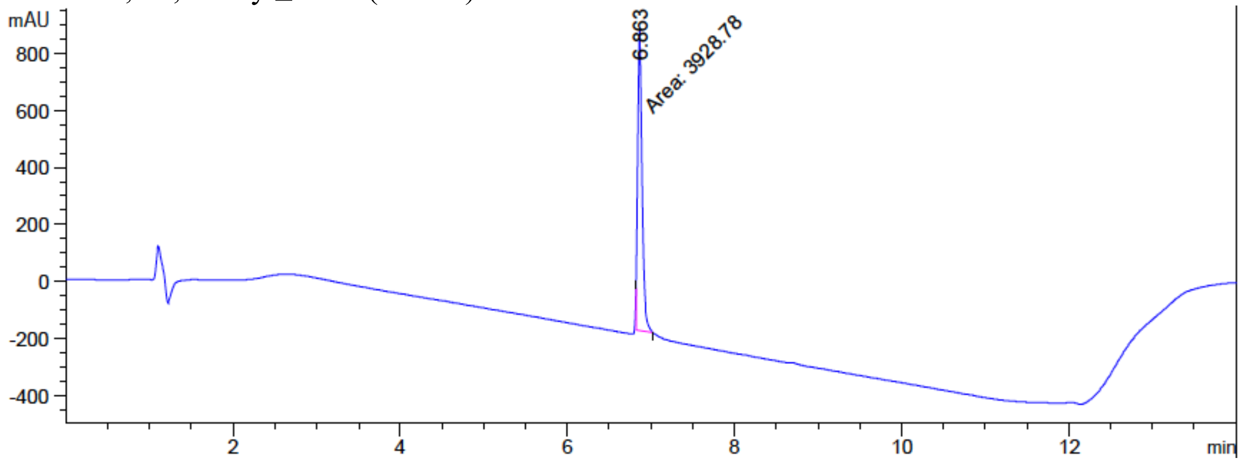

Method C, **25**, Purity ≥ 99% (MeOH)

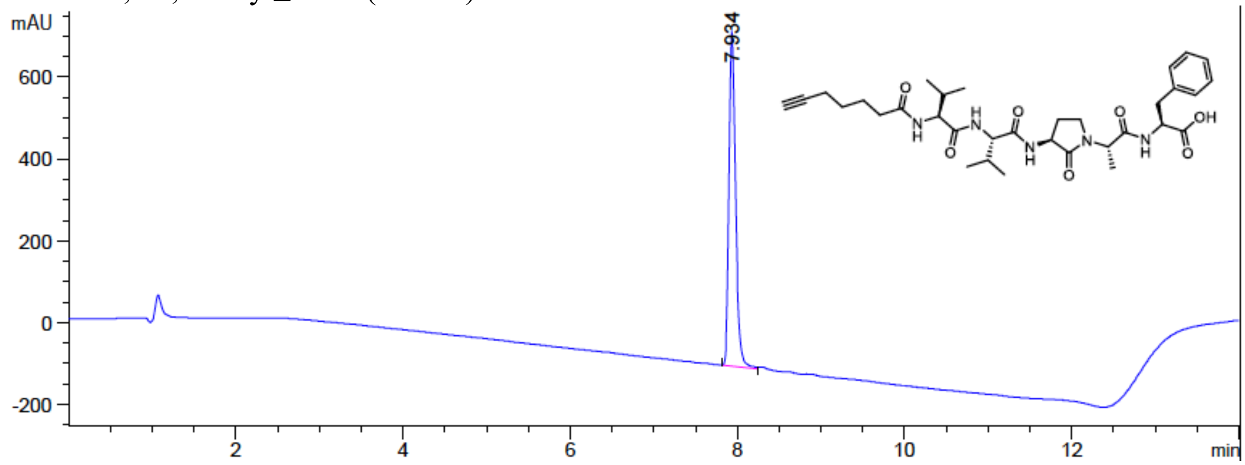

Method D, **25**, Purity ≥ 99% (MeCN)

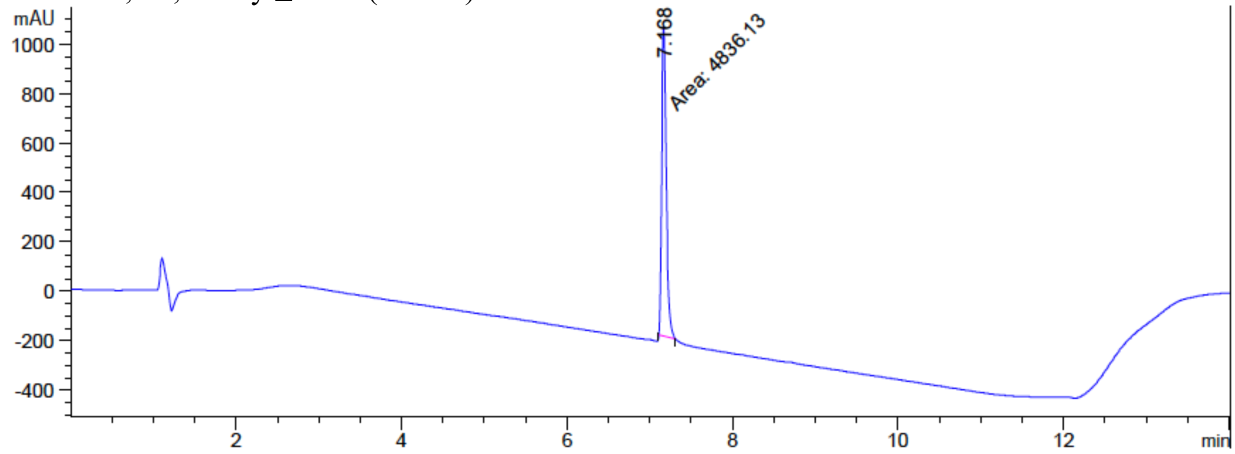

Method C, **26**, Purity ≥ 99% (MeOH)

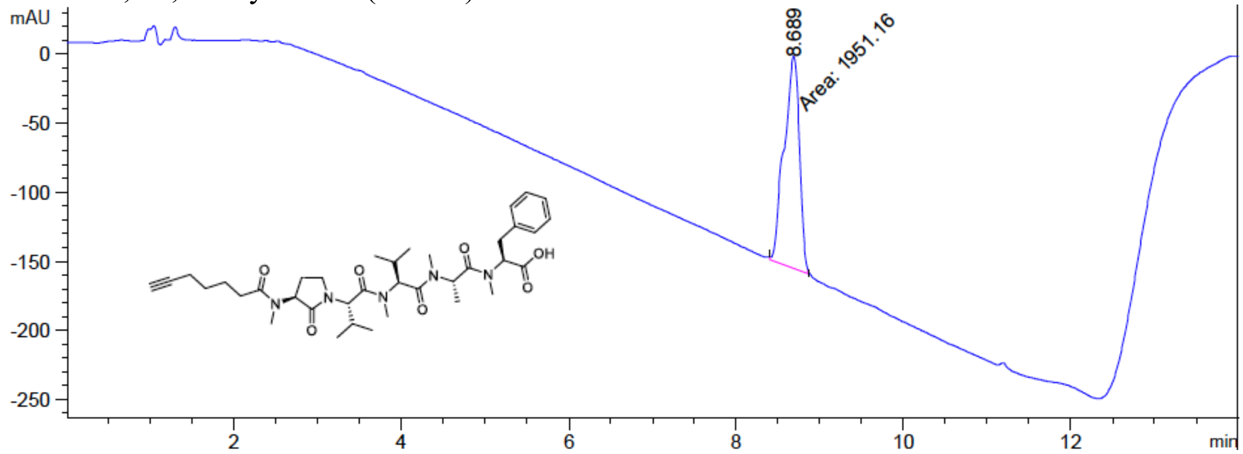

Method D, **26**, Purity ≥ 99% (MeCN)

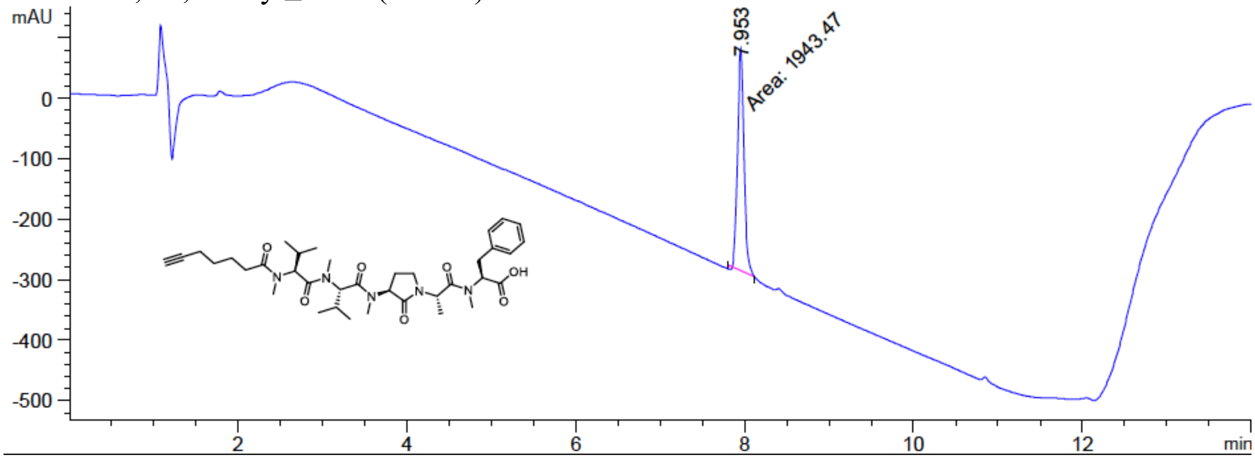

Method C, **27**, Purity ≥ 99% (MeOH)

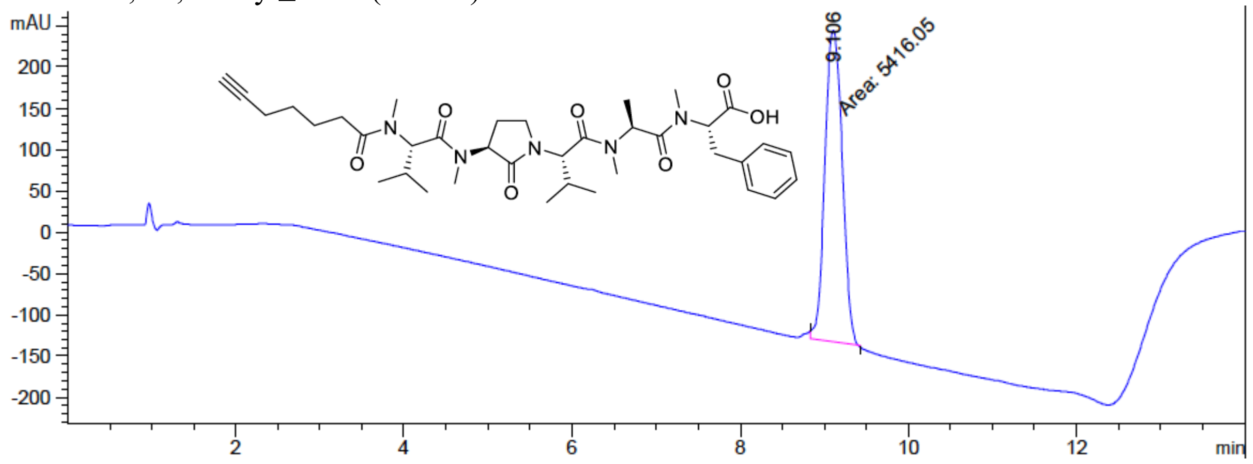

Method D, **27**, Purity ≥ 99% (MeCN)

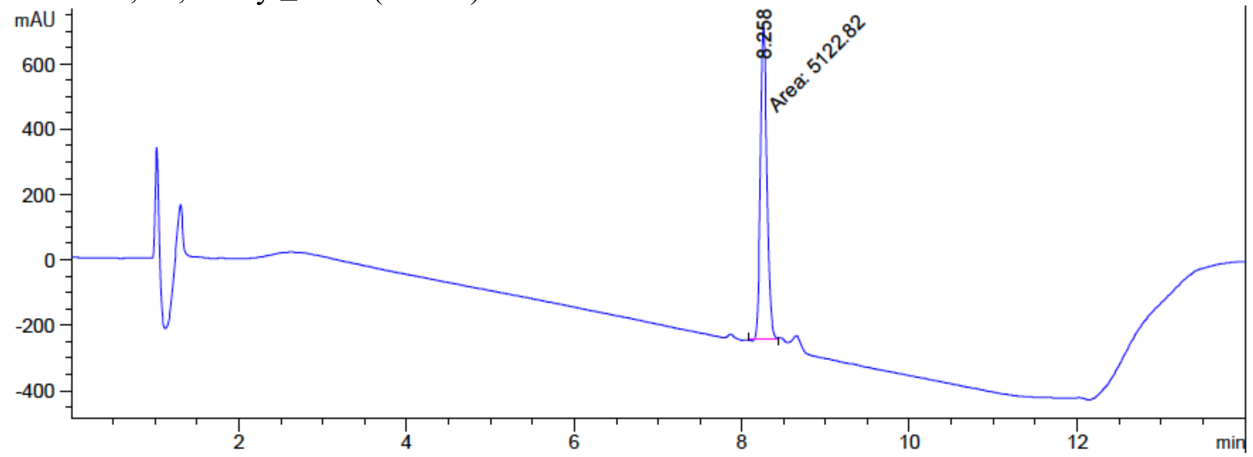

Method C, **28**, Purity ≥ 99% (MeOH)

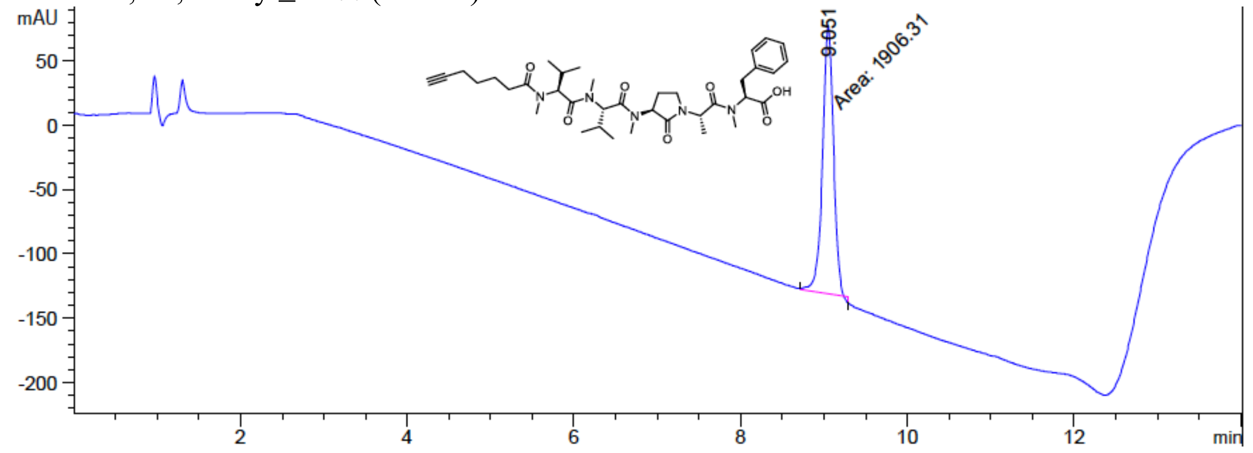

Method D, **28**, Purity ≥ 99% (MeCN)

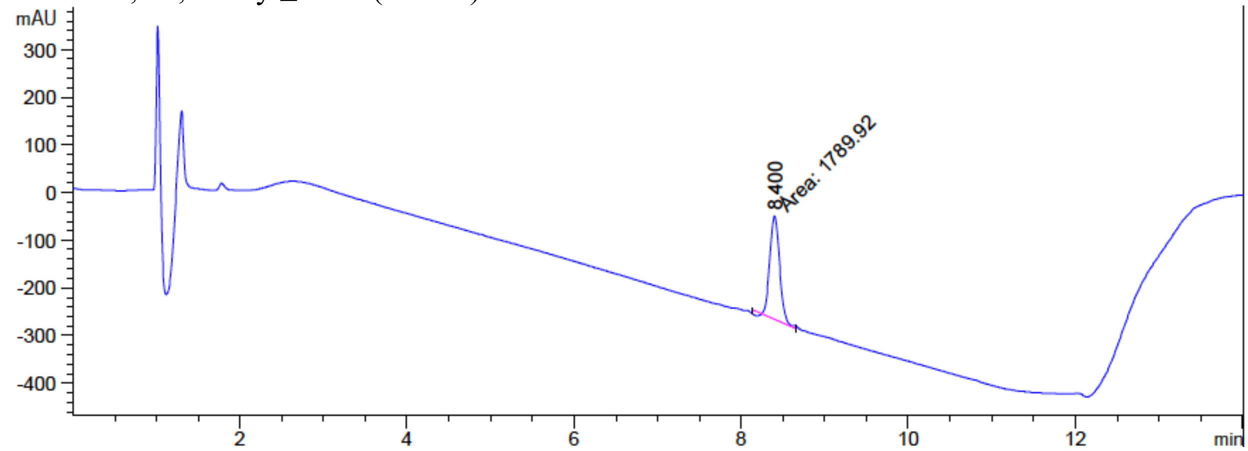

Method B, **29**, Purity ≥ 94% (MeOH)

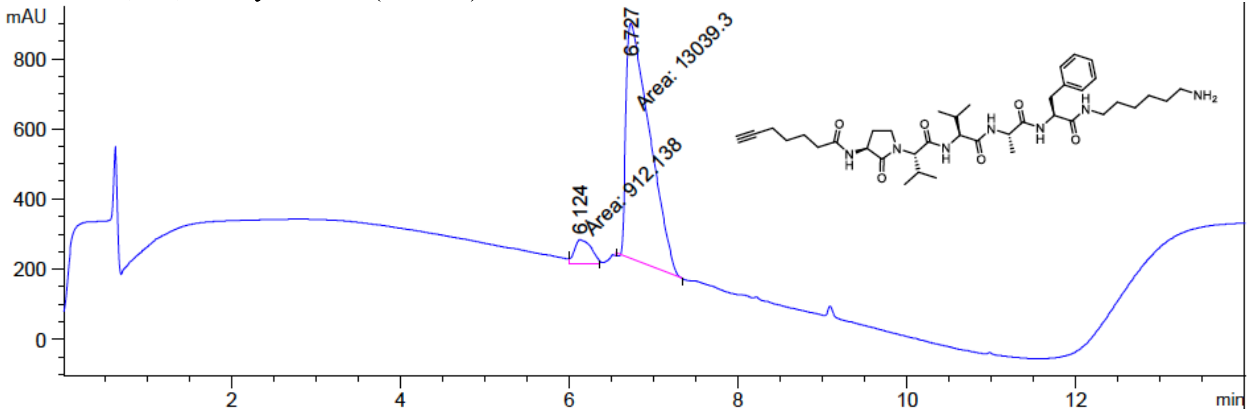

Method D, **29**, Purity ≥ 94% (MeCN)

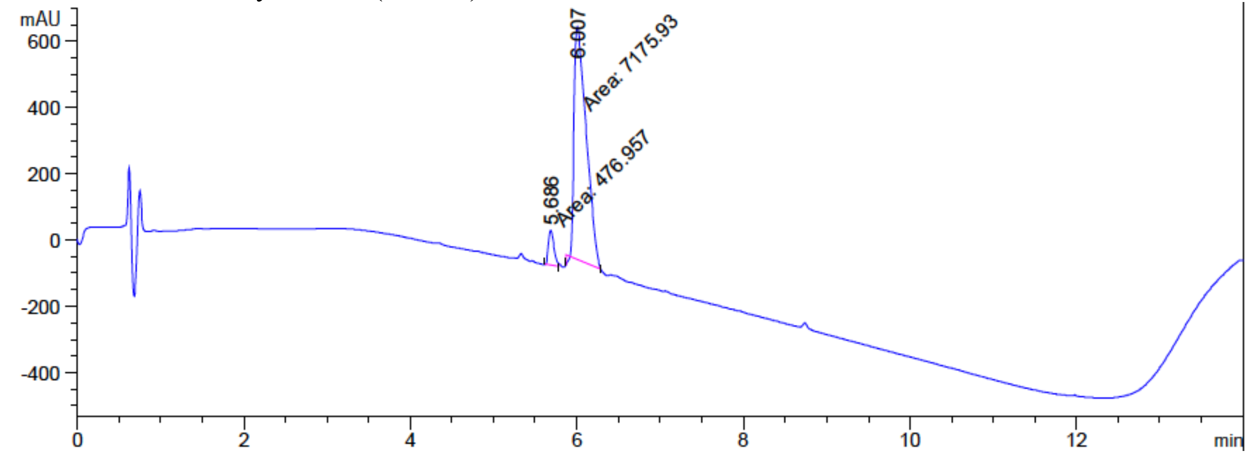

Method B, **30**, Purity ≥ 99% (MeOH)

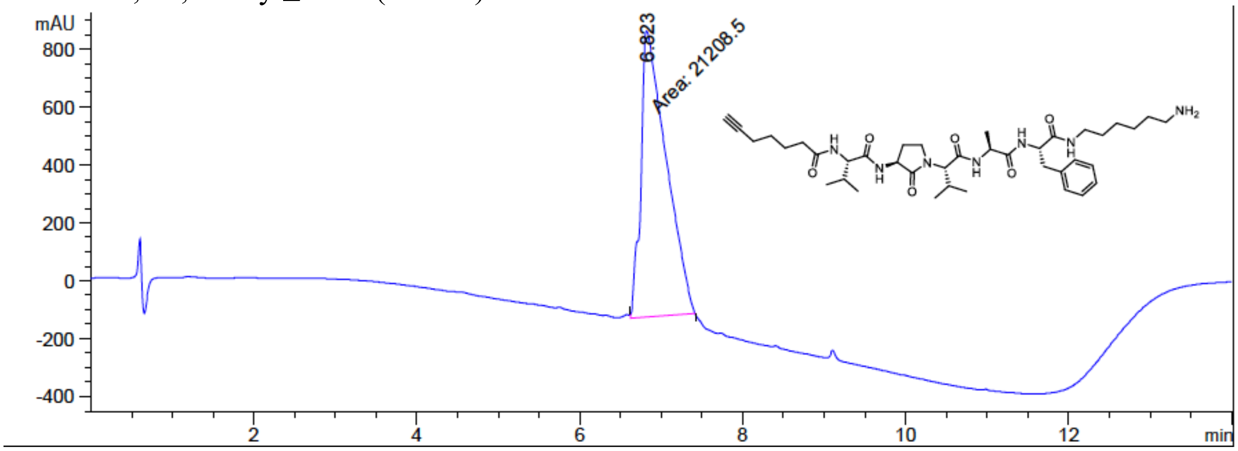

Method D, **30**, Purity ≥ 96% (MeCN)

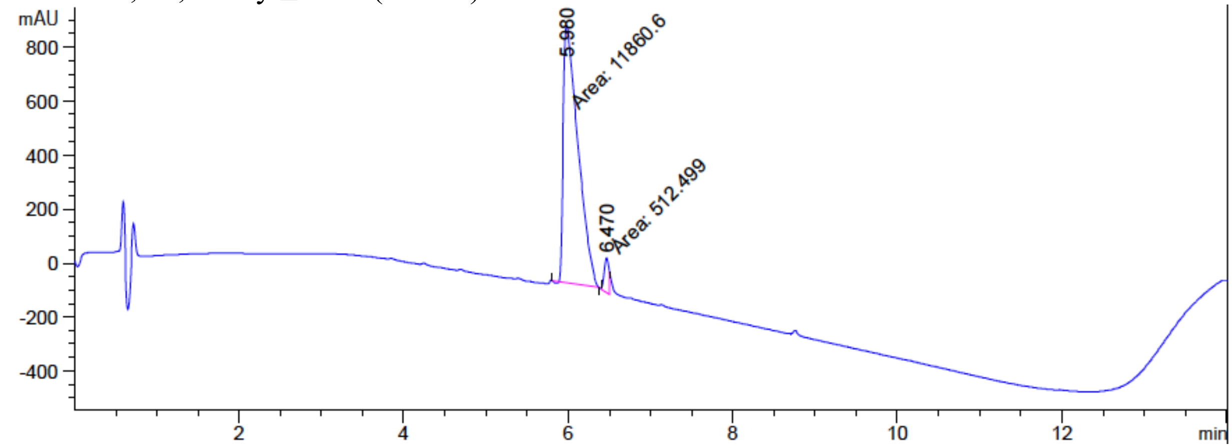

Method C, **31**, Purity ≥ 99% (MeOH)

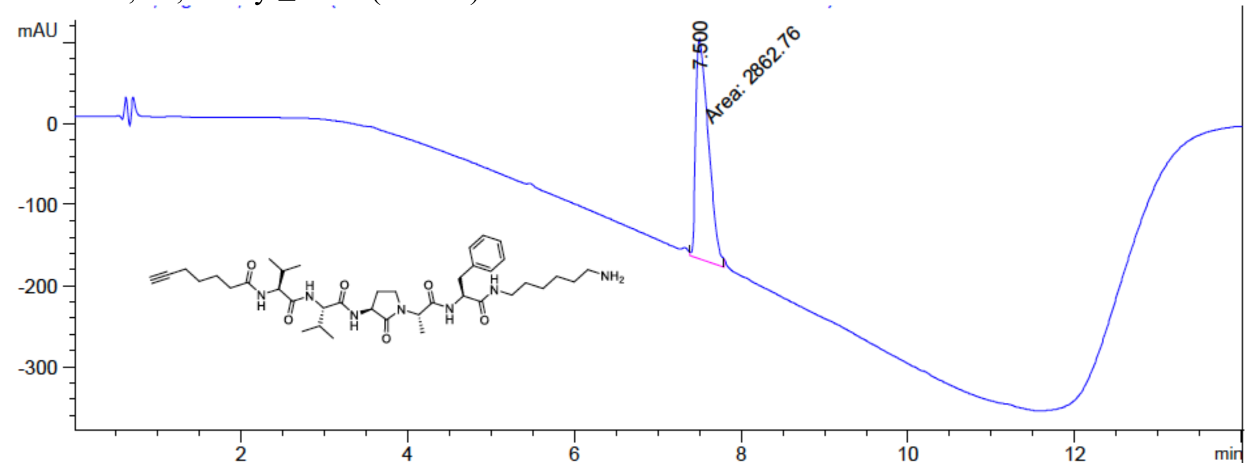

Method D, **31**, Purity ≥ 99% (MeCN)

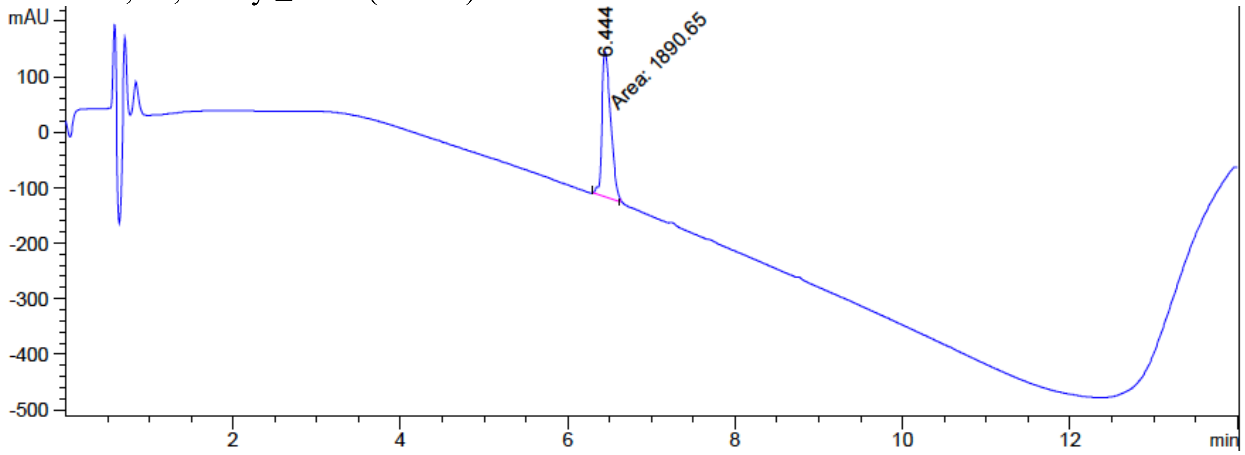

Method A, **32**, Purity ≥ 92% (MeOH)

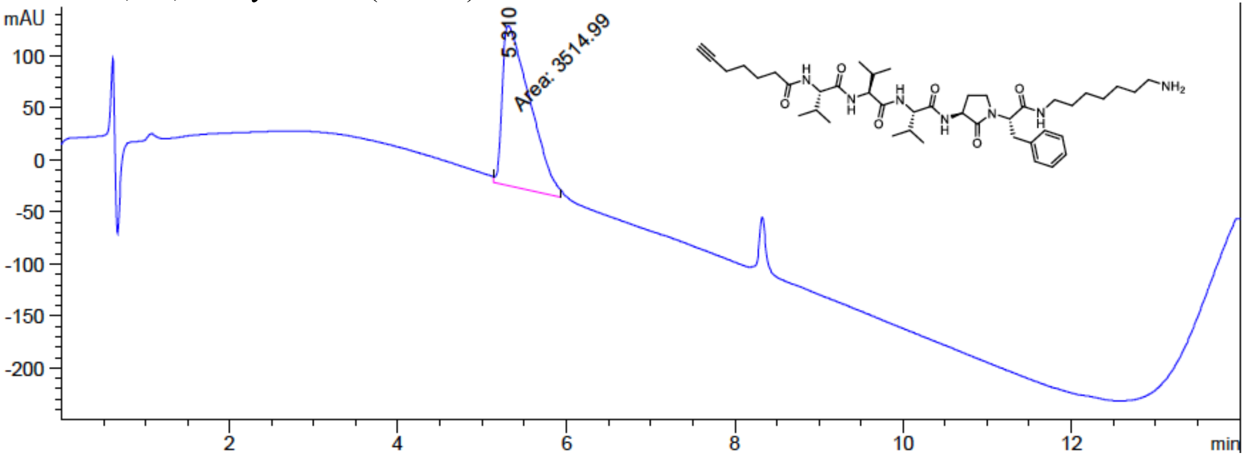

Method A, ***R*-32**, Purity ≥ 94% (MeOH)

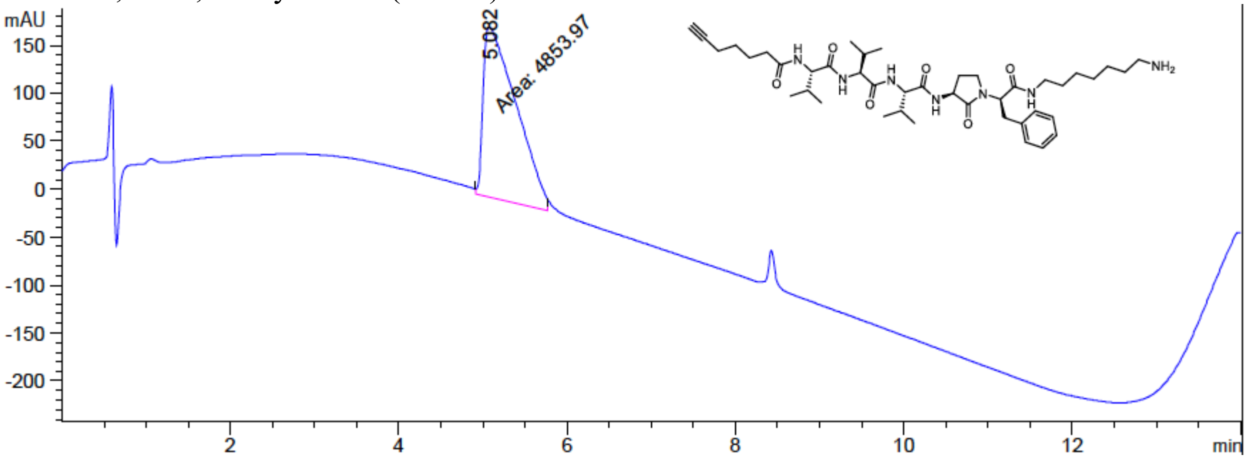

Method D, **32**, Purity ≥ 99% (MeCN)

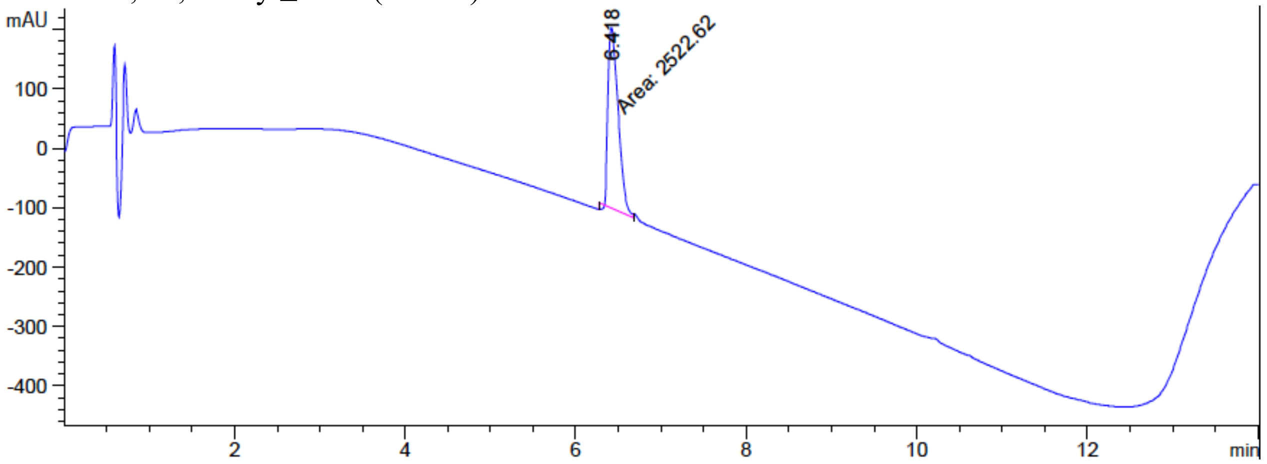

Method C, **33**, Purity ≥ 99% (MeOH)

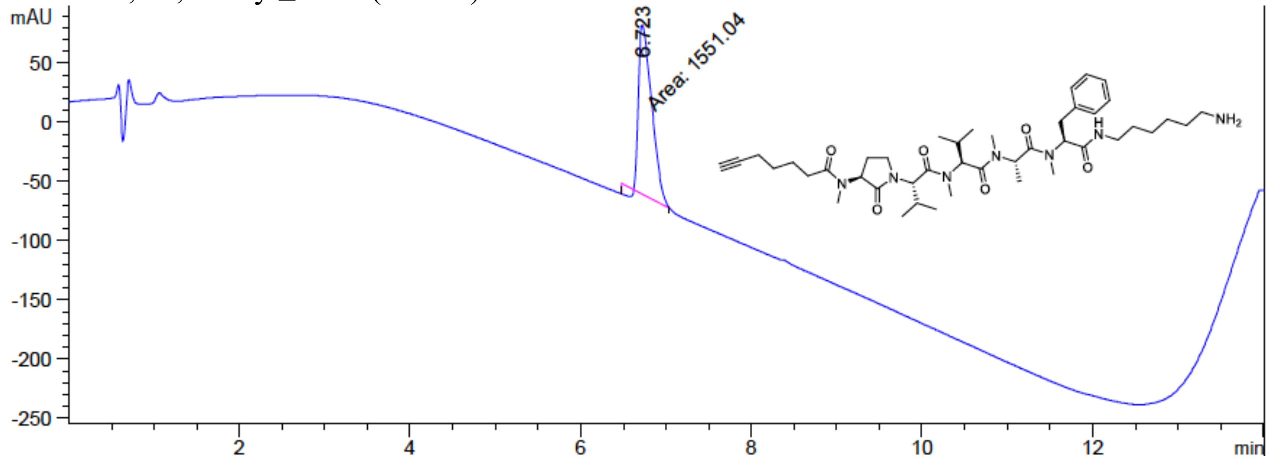

Method D, **33**, Purity ≥ 99% (MeCN)

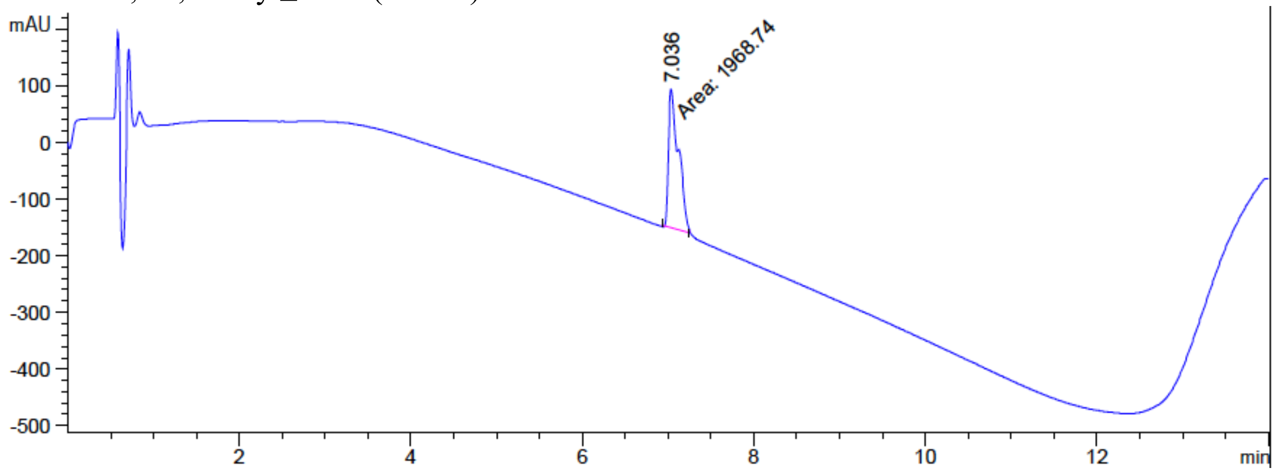

Method C, **34**, Purity ≥ 99% (MeOH)

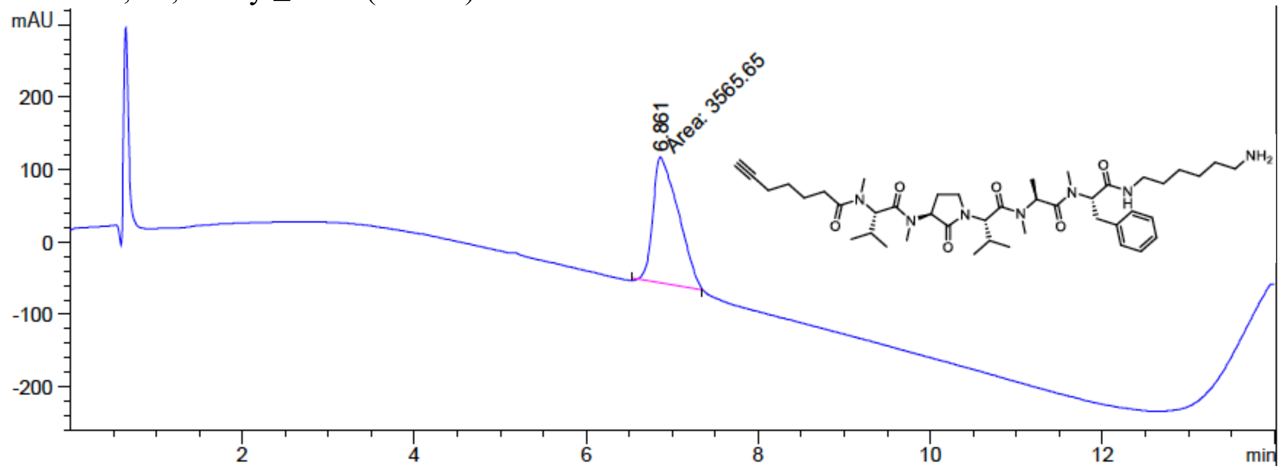

Method D, **34**, Purity ≥ 99% (MeCN)

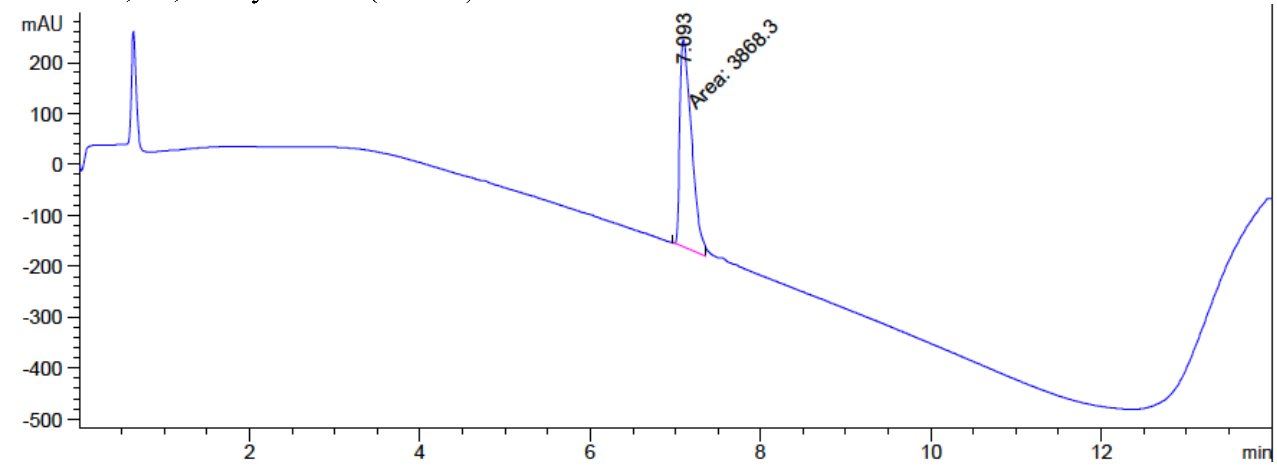

Method C, **35**, Purity ≥ 99% (MeOH)

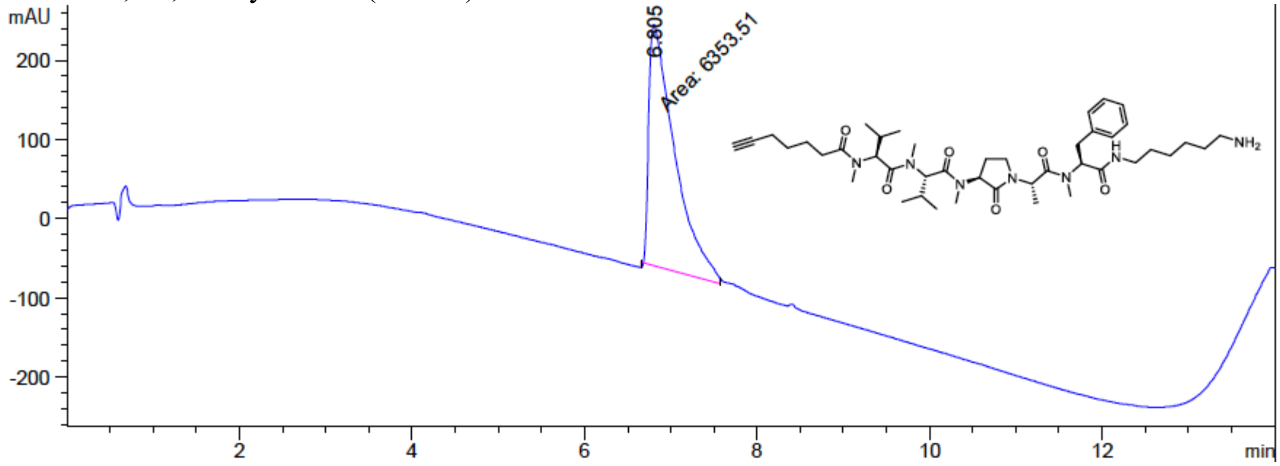

Method D, **35**, Purity ≥ 99% (MeCN)

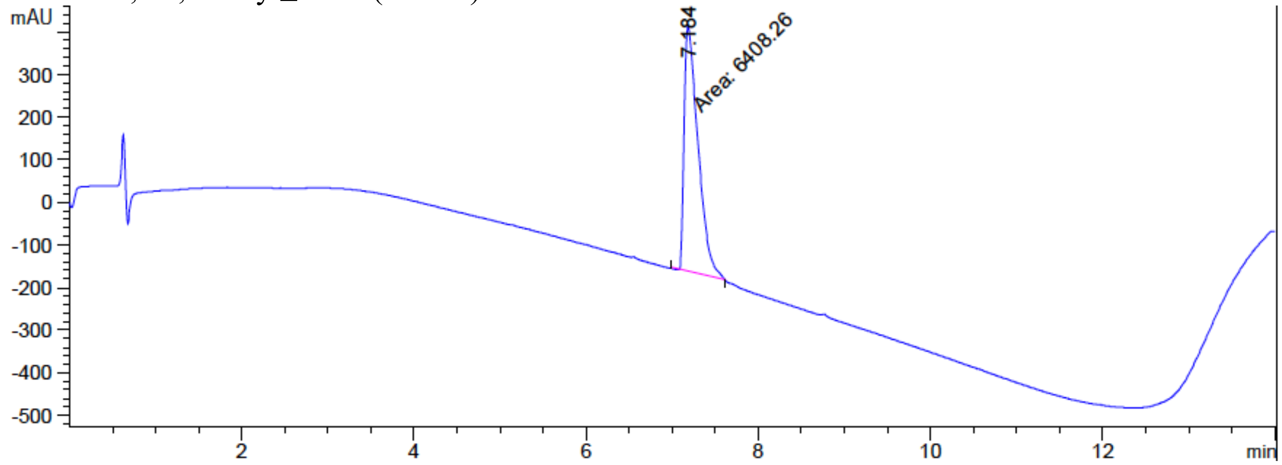

Method C, **36**, Purity ≥ 99% (MeOH)

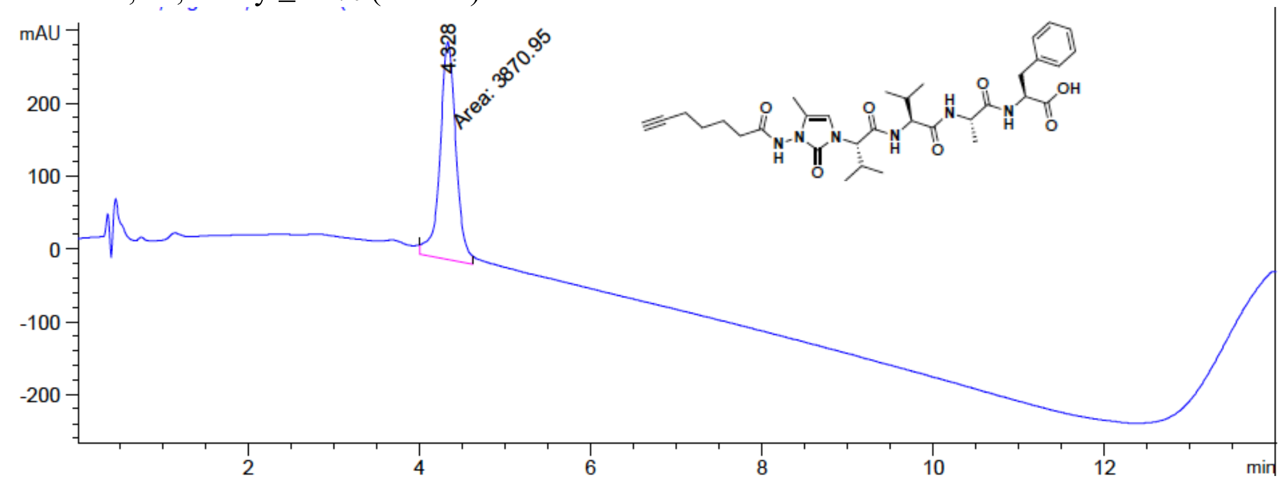

Method D, **36**, Purity ≥ 99% (MeCN)

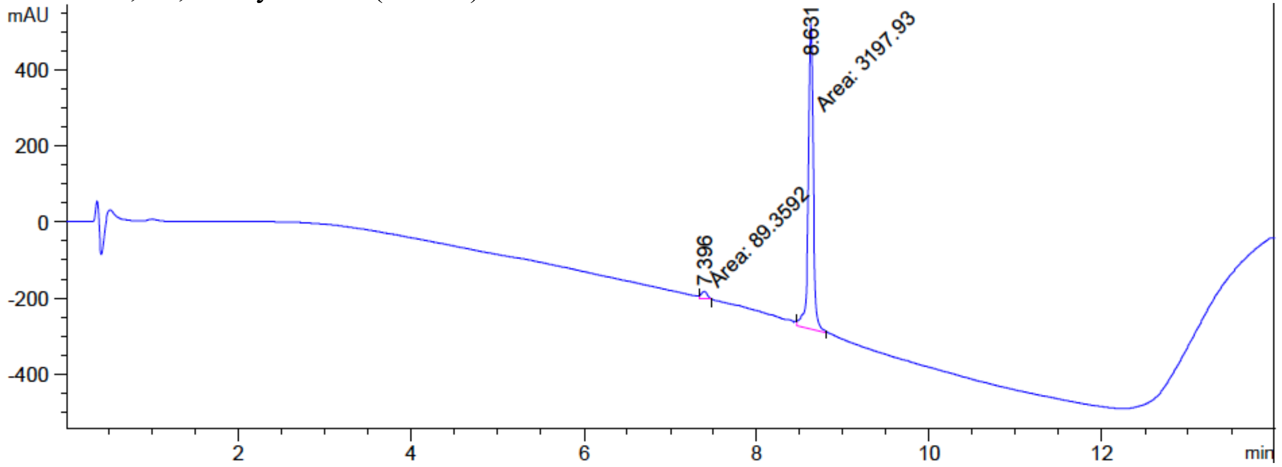

Method C, **37**, Purity ≥ 96% (MeOH)

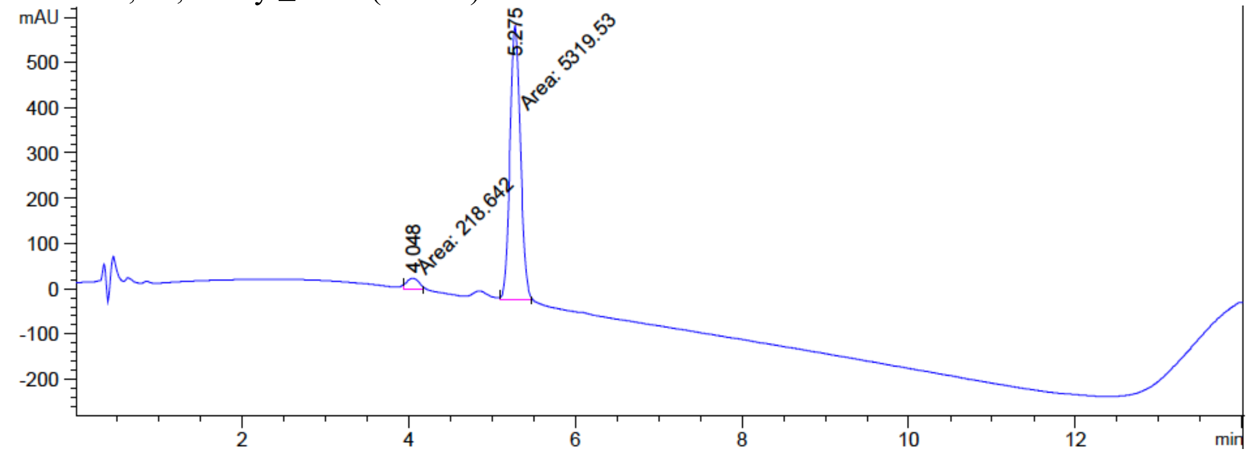

Method D, **37**, Purity ≥ 96% (MeCN)

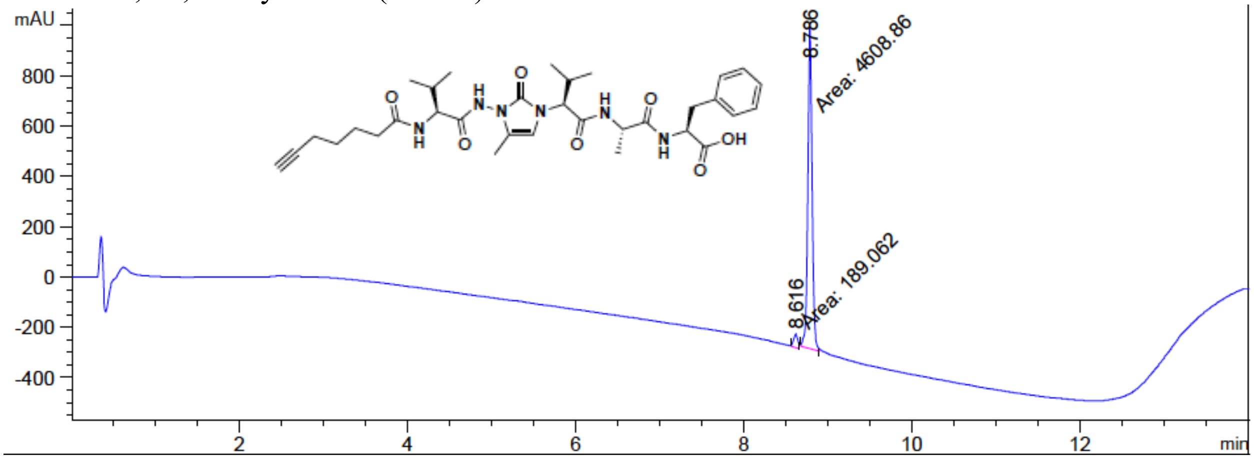

Dose-response curves of *Leishmania* strains and LM-1 macrophage survival rate in the presence of peptides 12-37

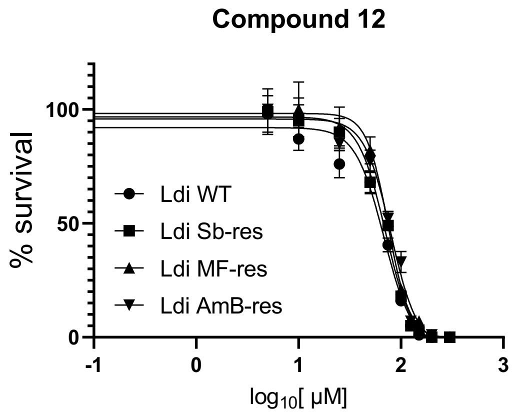

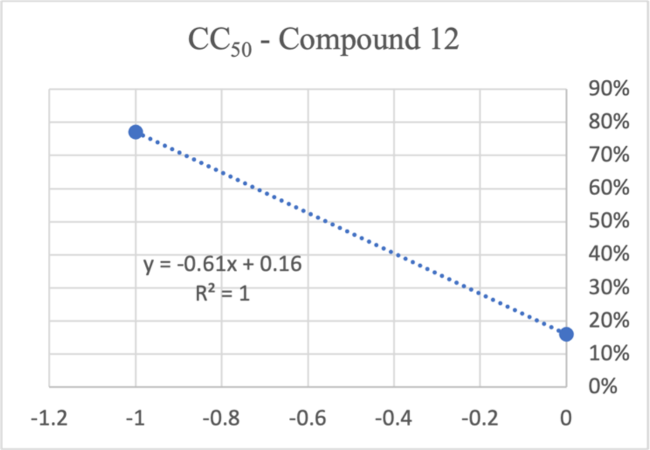

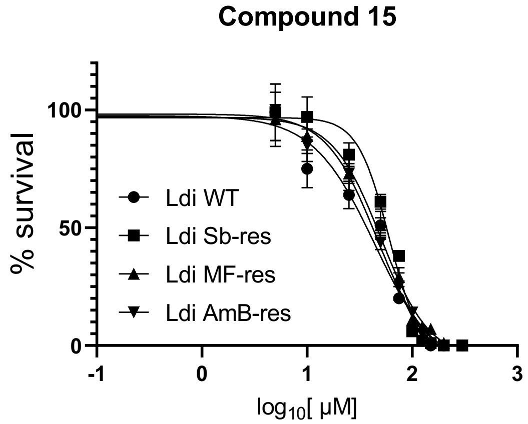

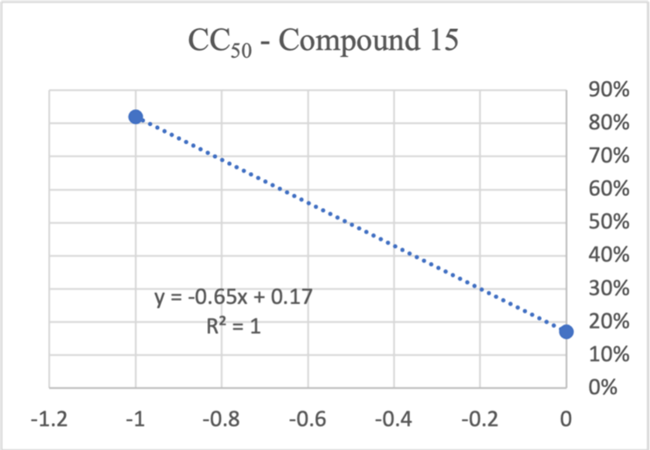

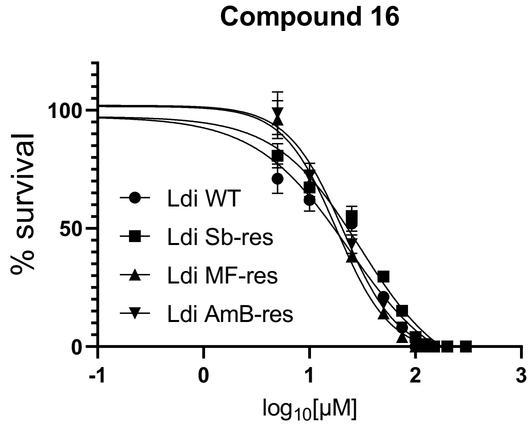

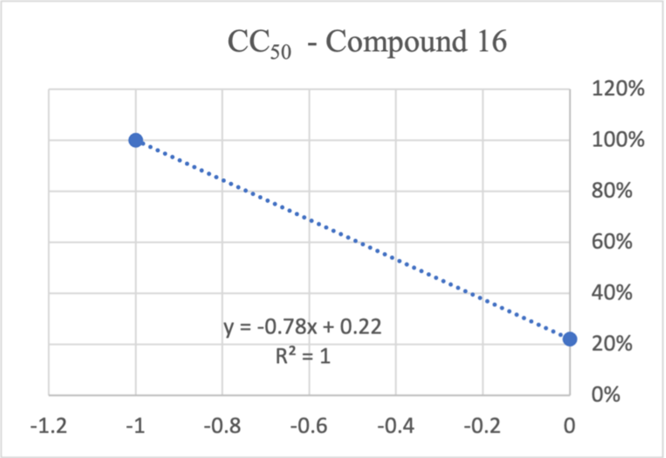

Compound 17

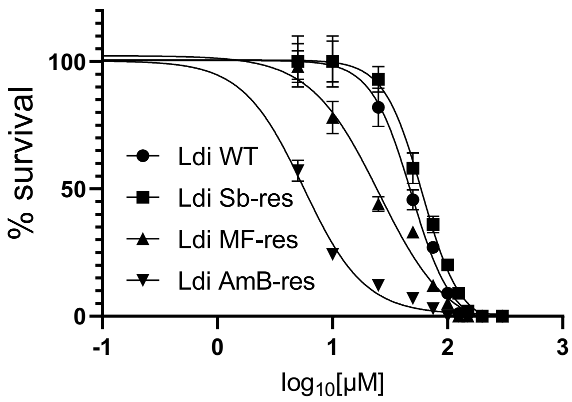

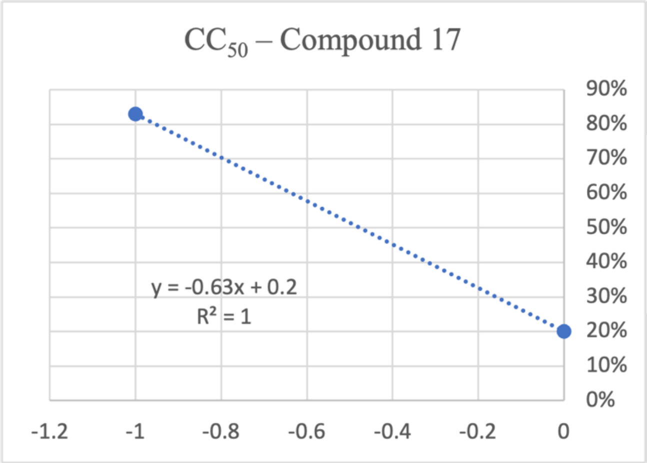

Compound 18

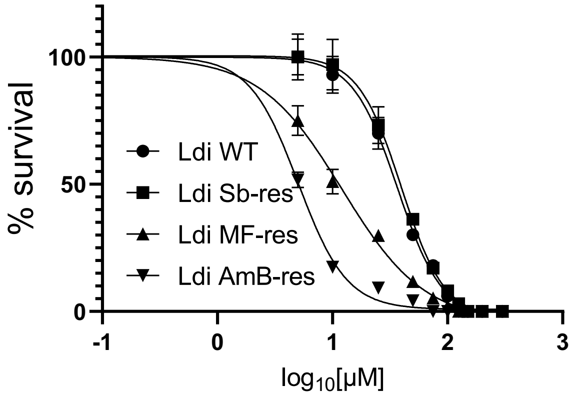

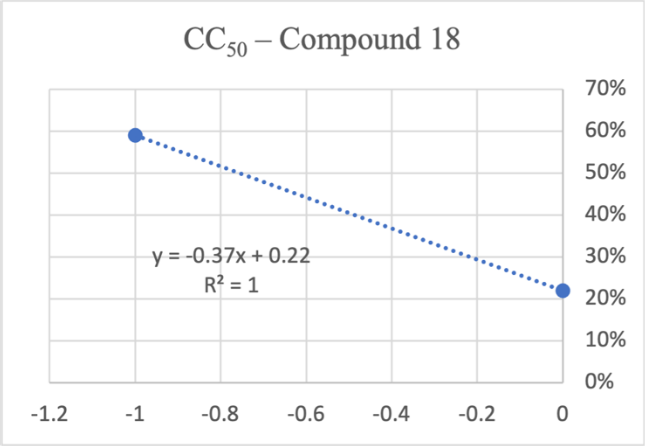

Compound 19

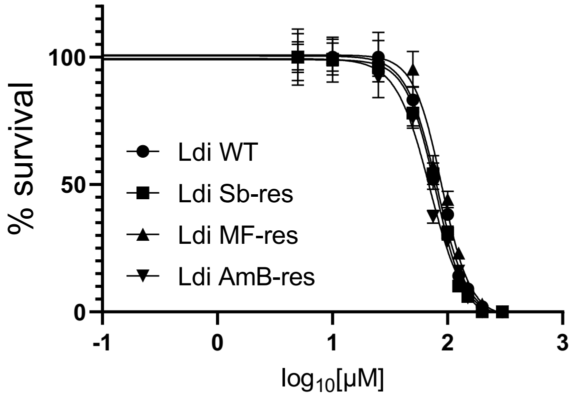

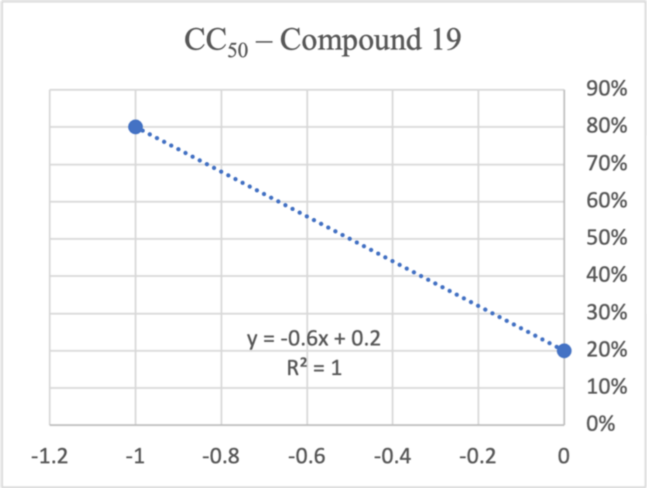

Compound 20

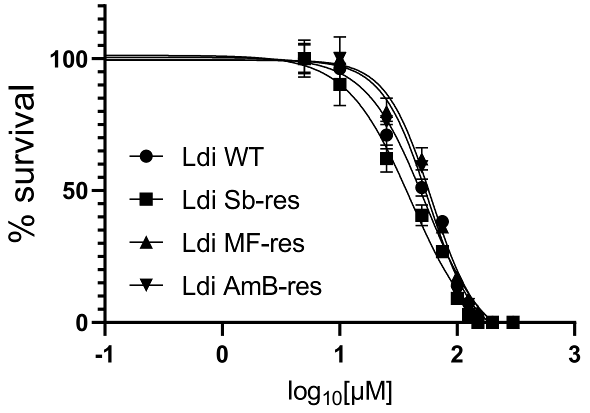

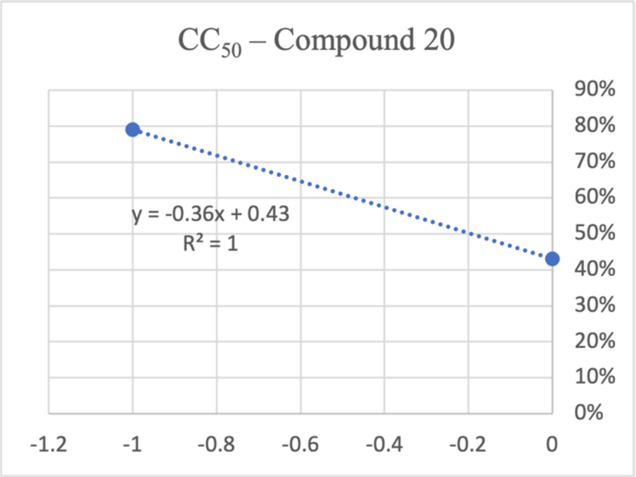

Compound 21

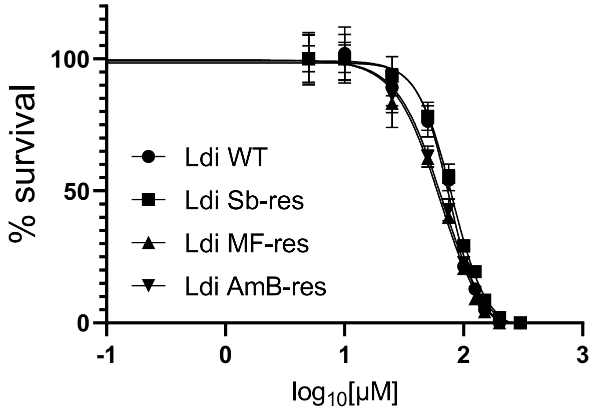

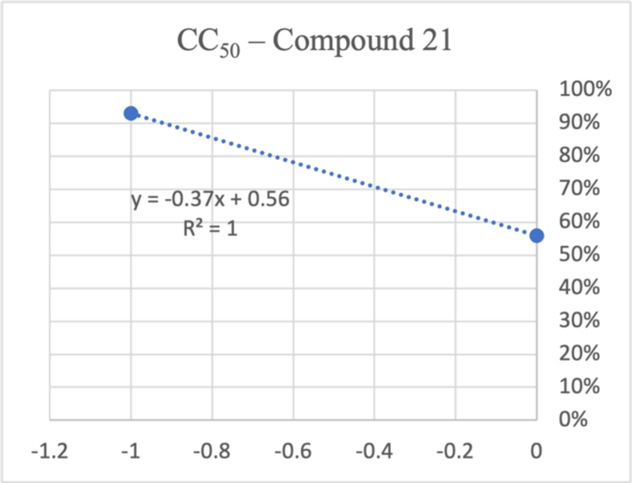

Compound 22

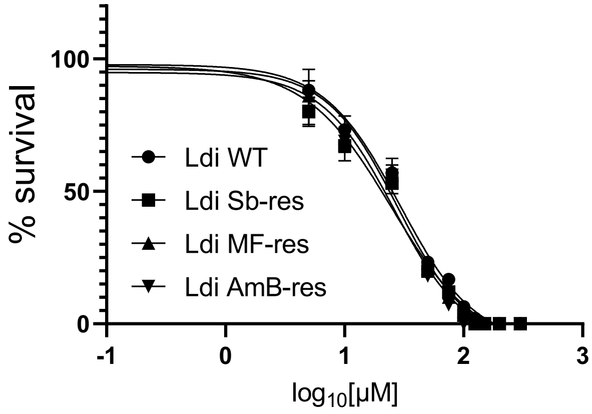

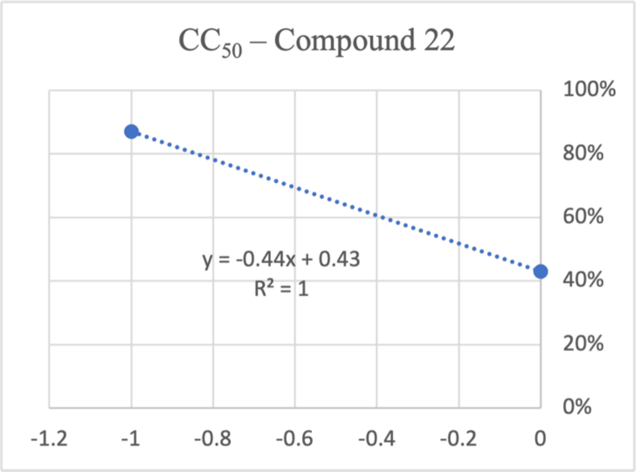

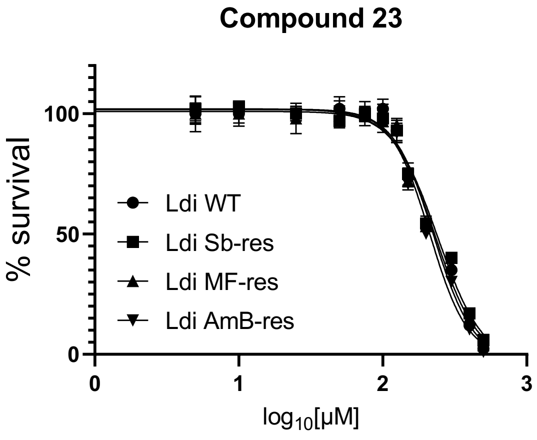
Compound 23
% survival
100
50
0
0
1
2
3
log10[μM]
Ldi WT
Ldi Sb-res
Ldi MF-res
Ldi AmB-res

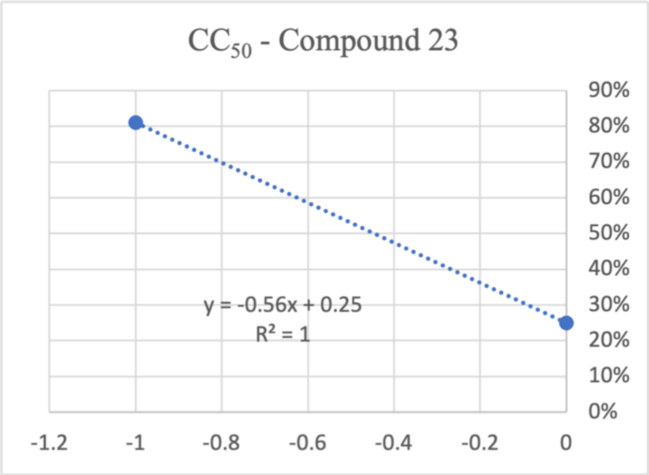
CC50 - Compound 23
y = -0.56x + 0.25
R² = 1
% survival
90%
80%
70%
60%
50%
40%
30%
20%
10%
0%
-1.2
-1
-0.8
-0.6
-0.4
-0.2
0

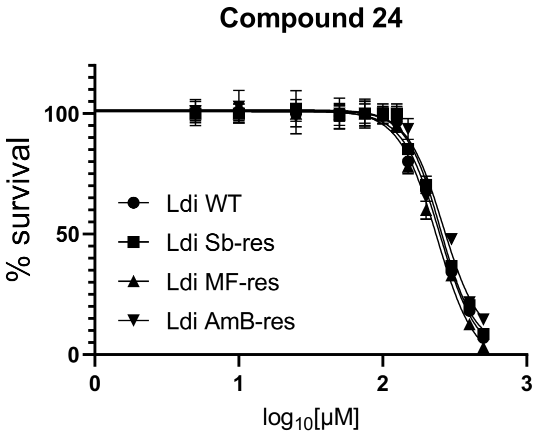
Compound 24
% survival
100
50
0
0
1
2
3
log10[μM]
Ldi WT
Ldi Sb-res
Ldi MF-res
Ldi AmB-res

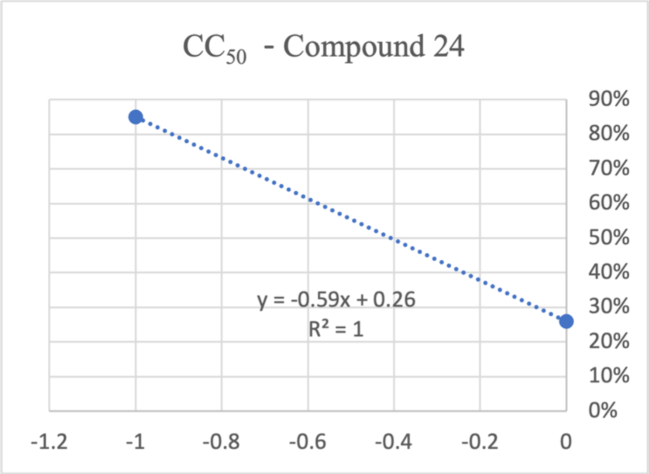
CC50 - Compound 24
y = -0.59x + 0.26
R² = 1
% survival
90%
80%
70%
60%
50%
40%
30%
20%
10%
0%
-1.2
-1
-0.8
-0.6
-0.4
-0.2
0

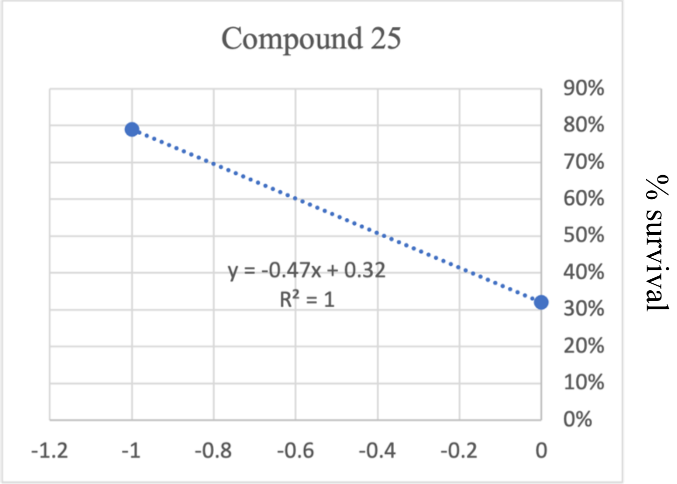
Compound 25
y = -0.47x + 0.32
R² = 1
% survival
90%
80%
70%
60%
50%
40%
30%
20%
10%
0%
-1.2
-1
-0.8
-0.6
-0.4
-0.2
0

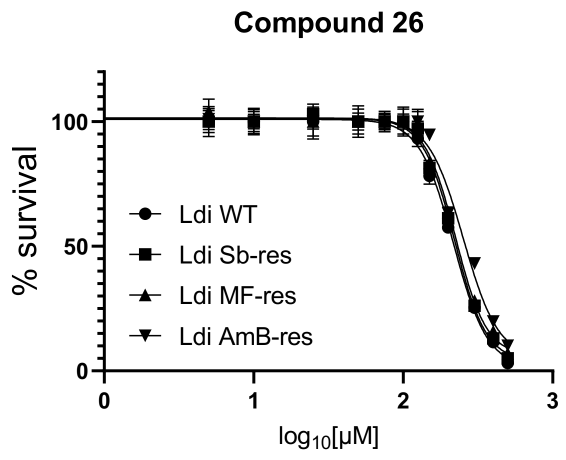
Compound 26
% survival
100
50
0
0
1
2
3
log10[μM]
Ldi WT
Ldi Sb-res
Ldi MF-res
Ldi AmB-res

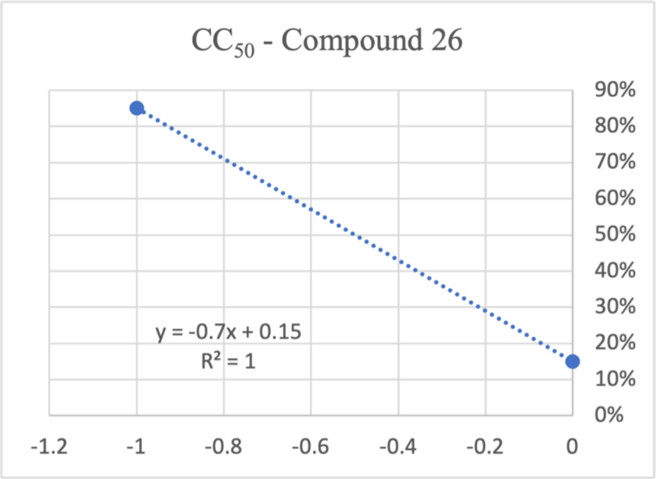
CC50 - Compound 26
y = -0.7x + 0.15
R² = 1
90%
80%
70%
60%
50%
40%
30%
20%
10%
0%
% survival
-1.2
-1
-0.8
-0.6
-0.4
-0.2
0

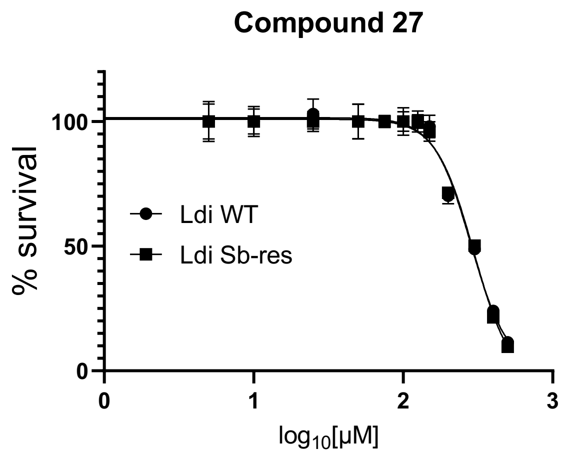
Compound 27
% survival
100
50
0
0
1
2
3
log10[μM]
Ldi WT
Ldi Sb-res

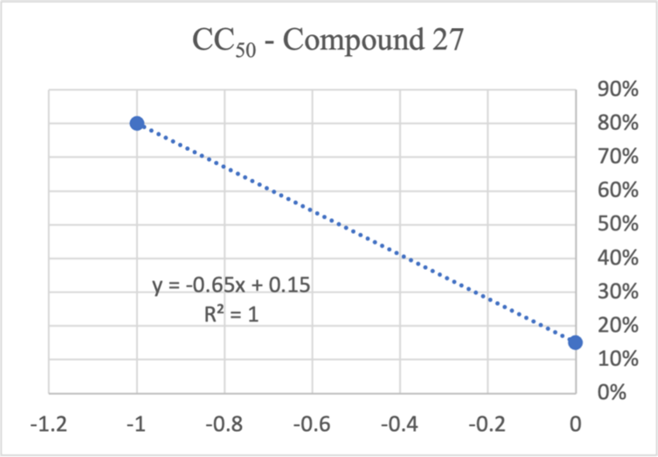
CC50 - Compound 27
y = -0.65x + 0.15
R² = 1
90%
80%
70%
60%
50%
40%
30%
20%
10%
0%
% survival
-1.2
-1
-0.8
-0.6
-0.4
-0.2
0

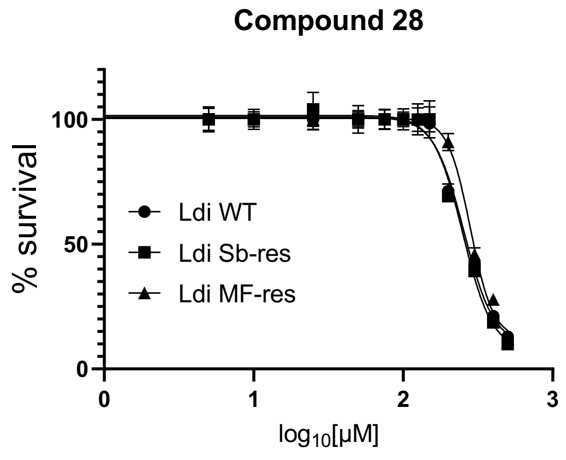
Compound 28
% survival
100
50
0
0
1
2
3
log10[μM]
Ldi WT
Ldi Sb-res
Ldi MF-res

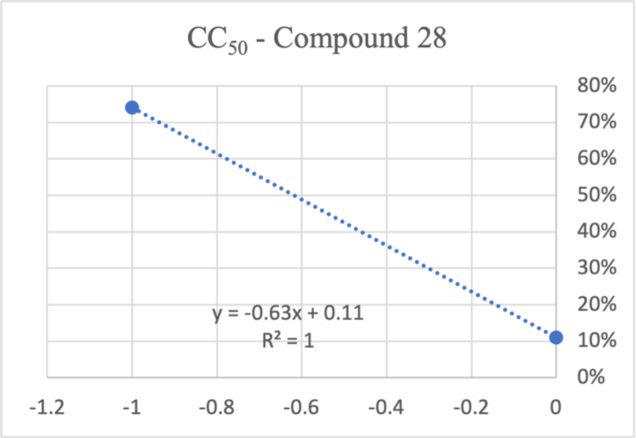
CC50 - Compound 28
y = -0.63x + 0.11
R² = 1
80%
70%
60%
50%
40%
30%
20%
10%
0%
% survival
-1.2
-1
-0.8
-0.6
-0.4
-0.2
0

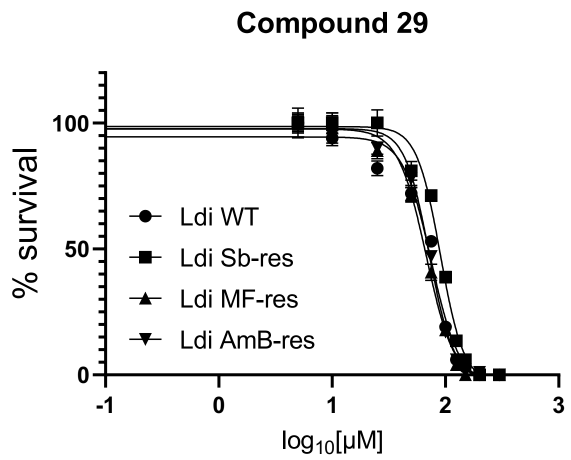
Compound 29
% survival
100
50
0
Ldi WT
Ldi Sb-res
Ldi MF-res
Ldi AmB-res
-1
0
1
2
3
log10[μM]

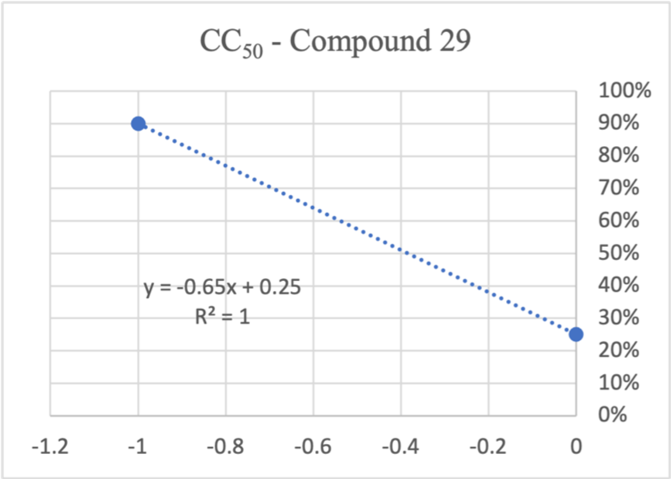
CC50 - Compound 29
y = -0.65x + 0.25
R² = 1
100%
90%
80%
70%
60%
50%
40%
30%
20%
10%
0%
% survival
-1.2
-1
-0.8
-0.6
-0.4
-0.2
0

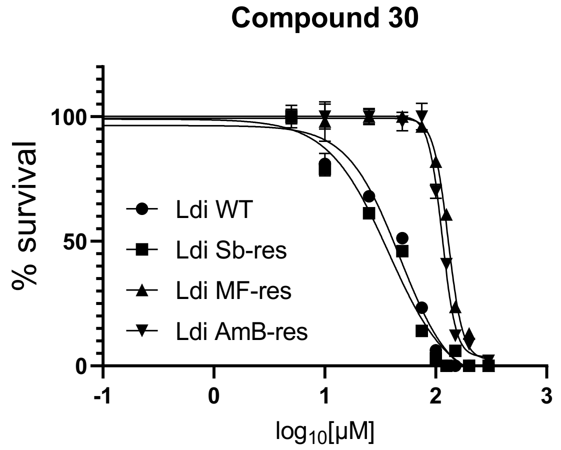
Compound 30
% survival
100
50
0
Ldi WT
Ldi Sb-res
Ldi MF-res
Ldi AmB-res
-1
0
1
2
3
log10[μM]

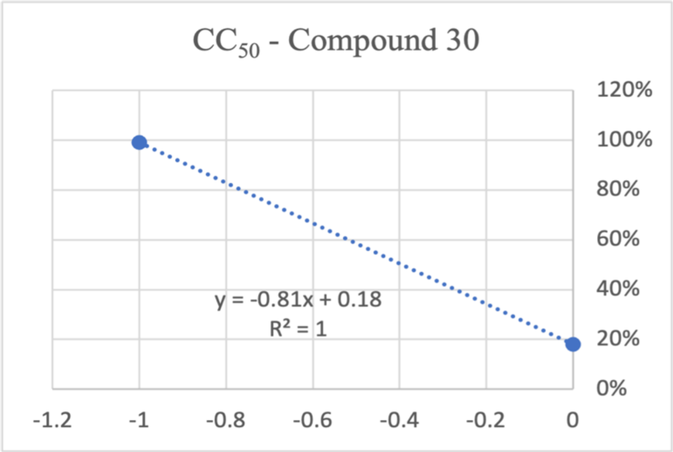
CC50 - Compound 30
y = -0.81x + 0.18
R² = 1
120%
100%
80%
60%
40%
20%
0%
% survival
-1.2
-1
-0.8
-0.6
-0.4
-0.2
0

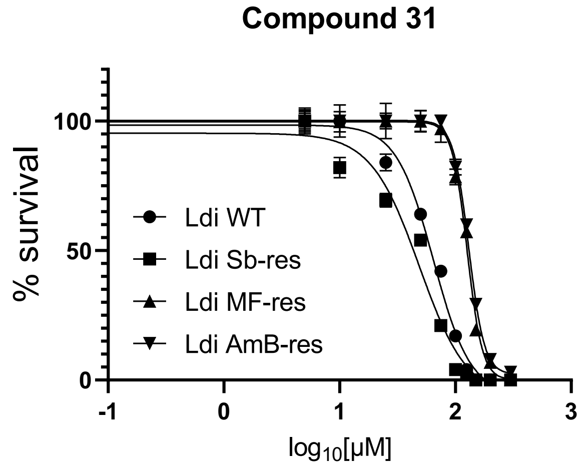
Compound 31
% survival
100
50
0
Ldi WT
Ldi Sb-res
Ldi MF-res
Ldi AmB-res
-1
0
1
2
3
log10[μM]

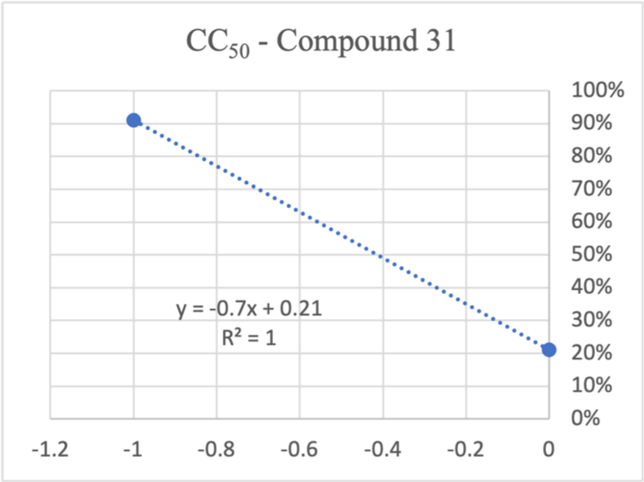
CC50 - Compound 31
y = -0.7x + 0.21
R² = 1
100%
90%
80%
70%
60%
50%
40%
30%
20%
10%
0%
% survival
-1.2
-1
-0.8
-0.6
-0.4
-0.2
0

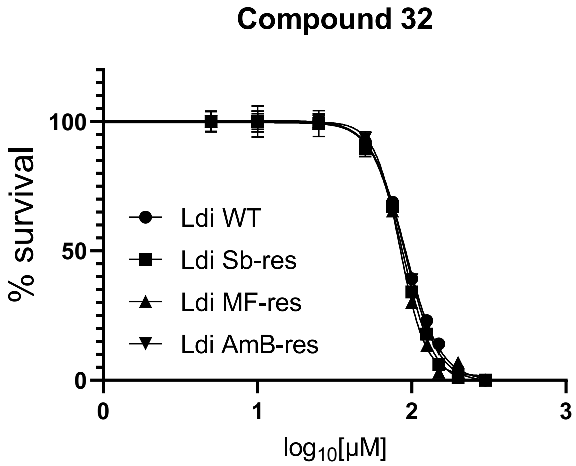
Compound 32
% survival
Ldi WT
Ldi Sb-res
Ldi MF-res
Ldi AmB-res
log10[μM]

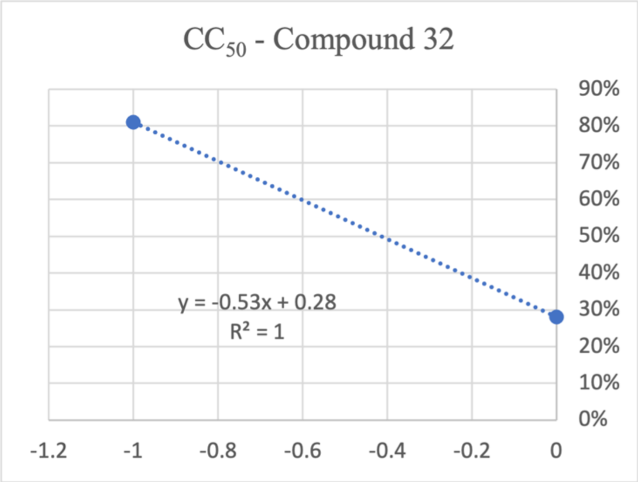
CC50 - Compound 32
y = -0.53x + 0.28
R² = 1
% survival

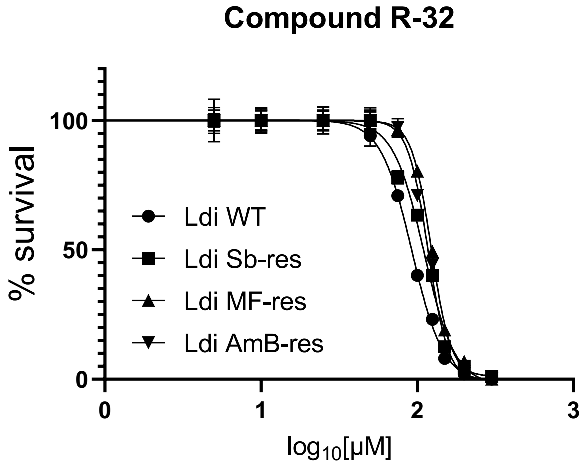
Compound R-32
% survival
Ldi WT
Ldi Sb-res
Ldi MF-res
Ldi AmB-res
log10[μM]

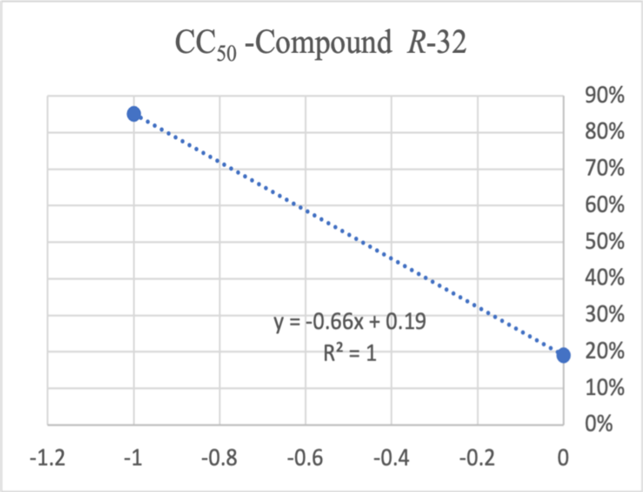
CC50 -Compound R-32
y = -0.66x + 0.19
R² = 1
% survival

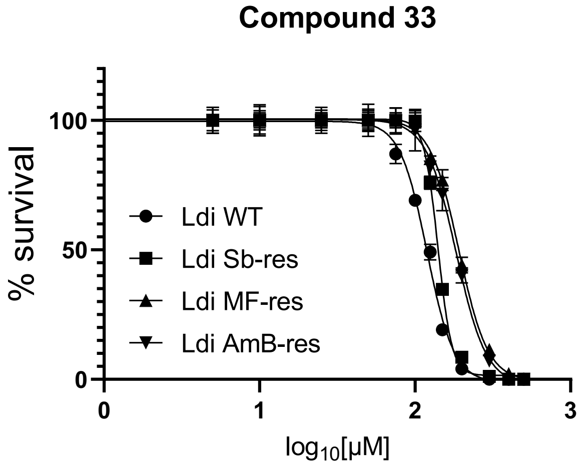
Compound 33
% survival
Ldi WT
Ldi Sb-res
Ldi MF-res
Ldi AmB-res
log10[μM]

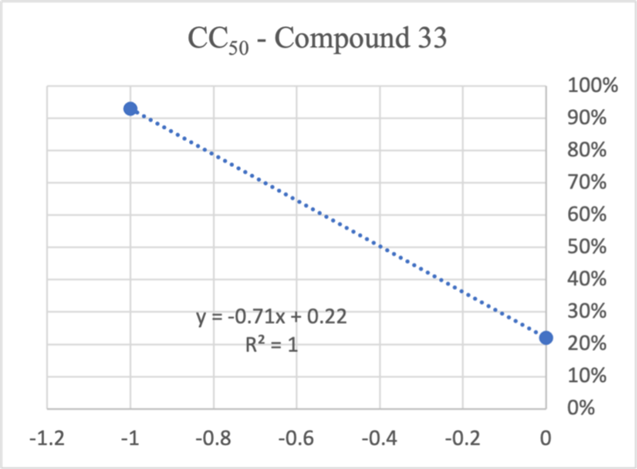
CC50 - Compound 33
y = -0.71x + 0.22
R² = 1
% survival

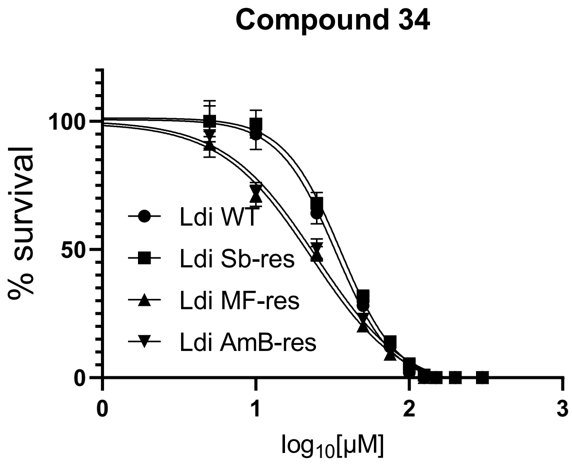
Compound 34
% survival
100
50
0
0
1
2
3
log10[µM]
Ldi WT
Ldi Sb-res
Ldi MF-res
Ldi AmB-res

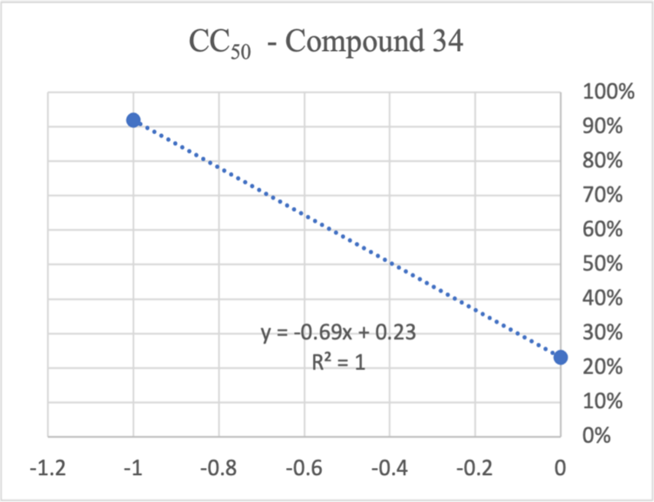
CC50 - Compound 34
y = -0.69x + 0.23
R² = 1
% survival
100%
90%
80%
70%
60%
50%
40%
30%
20%
10%
0%
-1.2
-1
-0.8
-0.6
-0.4
-0.2
0

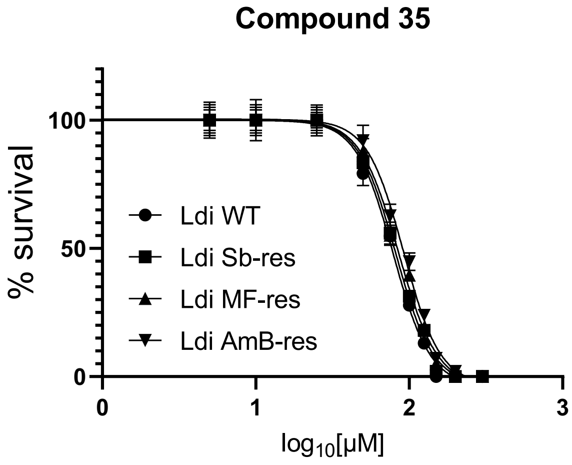
Compound 35
% survival
100
50
0
0
1
2
3
log10[µM]
Ldi WT
Ldi Sb-res
Ldi MF-res
Ldi AmB-res

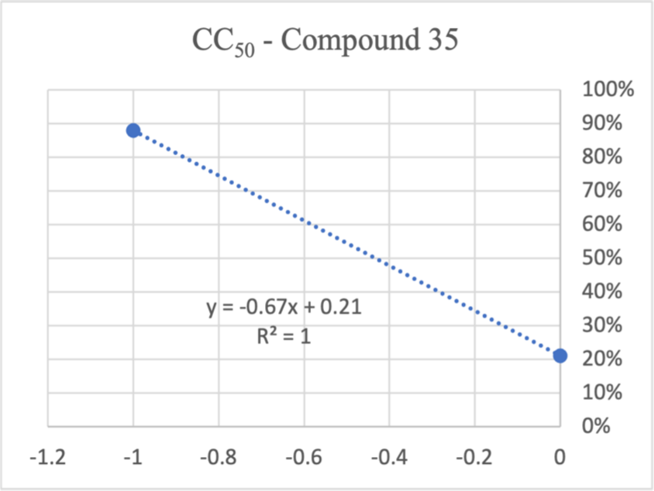
CC50 - Compound 35
y = -0.67x + 0.21
R² = 1
% survival
100%
90%
80%
70%
60%
50%
40%
30%
20%
10%
0%
-1.2
-1
-0.8
-0.6
-0.4
-0.2
0

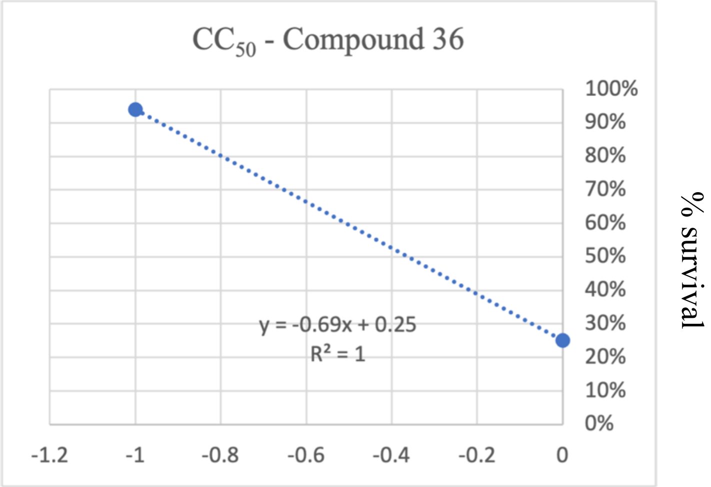
CC50 - Compound 36
y = -0.69x + 0.25
R² = 1
% survival
100%
90%
80%
70%
60%
50%
40%
30%
20%
10%
0%
-1.2
-1
-0.8
-0.6
-0.4
-0.2
0

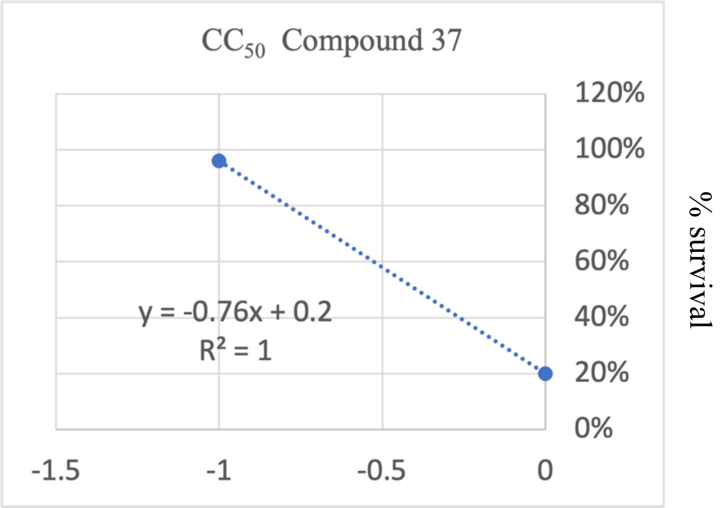
CC50 Compound 37
120%
100%
80%
60%
40%
20%
0%
% survival
y = -0.76x + 0.2
R² = 1
-1.5
-1
-0.5
0
